# Metabolic perturbations in mutants of glucose transporters and their applications in metabolite production in *Escherichia coli*

**DOI:** 10.1186/s12934-019-1224-8

**Published:** 2019-10-10

**Authors:** Hwi-Min Jung, Dae-Kyun Im, Jae Hyung Lim, Gyoo Yeol Jung, Min-Kyu Oh

**Affiliations:** 1 Department of Chemical and Biological Engineering, Korea University, 145 Anam-ro, Seongbuk-gu, Seoul, 02841 South Korea; 2 Department of Chemical Engineering, Pohang University of Science and Technology, 77 Cheongam-ro, Nam-gu, Pohang, Gyeongbuk 37673 South Korea; 3 School of Interdisciplinary Bioscience and Bioengineering, Pohang University of Science and Technology, 77 Cheongam-ro, Nam-gu, Pohang, Gyeongbuk 37673 South Korea

**Keywords:** Sugar transporters, Transcriptome analysis, ^13^C Metabolic flux analysis, Enhanced green fluorescent protein (EGFP), γ-Aminobutyrate (GABA), Lycopene

## Abstract

**Background:**

Most microorganisms have evolved to maximize growth rate, with rapid consumption of carbon sources from the surroundings. However, fast growing phenotypes usually feature secretion of organic compounds. For example, *E. coli* mainly produced acetate in fast growing condition such as glucose rich and aerobic condition, which is troublesome for metabolic engineering because acetate causes acidification of surroundings, growth inhibition and decline of production yield. The overflow metabolism can be alleviated by reducing glucose uptake rate.

**Results:**

As glucose transporters or their subunits were knocked out in *E. coli*, the growth and glucose uptake rates decreased and biomass yield was improved. Alteration of intracellular metabolism caused by the mutations was investigated with transcriptome analysis and ^13^C metabolic flux analysis (^13^C MFA). Various transcriptional and metabolic perturbations were identified in the sugar transporter mutants. Transcription of genes related to glycolysis, chemotaxis, and flagella synthesis was downregulated, and that of gluconeogenesis, Krebs cycle, alternative transporters, quorum sensing, and stress induced proteins was upregulated in the sugar transporter mutants. The specific production yields of value-added compounds (enhanced green fluorescent protein, γ-aminobutyrate, lycopene) were improved significantly in the sugar transporter mutants.

**Conclusions:**

The elimination of sugar transporter resulted in alteration of global gene expression and redirection of carbon flux distribution, which was purposed to increase energy yield and recycle carbon sources. When the pathways for several valuable compounds were introduced to mutant strains, specific yield of them were highly improved. These results showed that controlling the sugar uptake rate is a good strategy for ameliorating metabolite production.

## Background

Microorganisms have evolved with cooperation and competition in the ecosystem. In a microbial consortium, the bacteria that have fast-growing phenotypes are at an advantage to occupy their surroundings. Fast dividing organisms consume carbon substrate rapidly to generate energy for biomass formation. Interestingly, it was discovered that *Saccharomyces cerevisiae* is more prone to carrying out ethanol fermentation during growth in aerobic conditions, than its predecessors [1]. They rapidly consume glucose and accumulate ethanol, which is toxic to most other microorganisms. Later, they re-consume ethanol for further growth [2]. Theoretically, glucose can be completely oxidized to CO_2_ with production of much more ATPs from respiration than those from fermentation metabolism through glycolysis. Nevertheless, they have evolved and opted for the ethanol fermentation pathway because it is faster and easier than the lengthy process of respiration. Many researchers have observed the overproduction of organic compounds such as acetate, lactate or ethanol during aerobic growth of fast growing organisms, and suggested an “overflow metabolism” theory (i.e. Crabtree effect in yeast, acetate overflow in *E. coli*, and Warburg effect in cancer cell) [3–5]. Recently, the relationship between growth rate and acetate overflow was accurately predicted through flux balance analysis and thermodynamic modeling approaches in *E. coli* [3, 6]. Moreover, the overflow was also interpreted through real estate hypothesis, which implies that the surface to volume ratio of the cell is critical for overflow [7].

Fast growing bacteria have received attention in the bioprocess industry because of their ease of handling and economic feasibility [8, 9]. Despite its merits, there are some problems that have to be overcome for utilizing fast growing strains, such as excessive formation of by-products [7, 10]. Formation of organic acids as by-products is accompanied by acidification of the culture broth, lowered biomass yield, and inefficient energy generation. In this aspect, a fast-growing phenotype, which is beneficial for natural selection, is not always favorable in the engineering of microorganisms. Slow but efficient growth could be helpful for pure cultures in sterilized fermenters, where the cell to cell competition is absent. The substrate uptake capability is closely related to the growth rate of microbes and furthermore, affects the acetate overflow. When adapted in a glucose-limited chemostat with aerobic conditions, *Saccharomyces cerevisiae* showed decrease in growth rate, but with delayed production of ethanol [11]. Similar phenomenon was observed in *E. coli*; when cultivated in low glucose condition, growth rate dwindled and little acetate was produced [12]. Additionally, non-PTS sugars could be consumed simultaneously in glucose limited condition [13].

Several major glucose uptake pathways have been determined in *E. coli*. Phosphotransferase system (PTS), by which glucose is transported and phosphorylated simultaneously, is an innate mechanism in almost all enteric bacteria [14]. The PTS affects diverse metabolism such as glycolysis, TCA cycle, acetate metabolism, respiration, etc., by modulating the intracellular concentration of cyclic AMP (cAMP) [15]. Additionally, glucose can be imported by non-specific transporters like *mglABC* (methyl-galactoside transport system), *malEFG* (maltose/maltodextrin transport system), and *galP* (galactose: H^+^ symporter) in *E. coli* [16]. When the major glucose uptake pathways were eliminated, the mutant showed declined growth rate, glucose uptake rate, and acetate production, which resembled the results of a glucose-limited chemostat study [17]. Theses physiological changes could be originated not only from slowing down of substrate uptake rate but also from perturbation of global metabolic networks. However, studies involving metabolism and its regulation by eliminating glucose uptake pathways are still lacking. Recently, omics technologies, such as transcriptomics or fluxomics, have been applied to investigate global perturbations in metabolic pathways by biochemical and environmental changes [18–21].

In this study, the glucose uptake in *E. coli* was impeded by removing major glucose transporters. As expected, retardation of growth was observed, little amount of acetate was produced, and biomass yield was improved in the sugar transporter mutants. Transcriptome analysis and ^13^C metabolic flux analysis (MFA) was implemented to compare the global gene expression and carbon flux changes. The phenotypic alterations of the sugar transporter mutants are speculated to help in improving the yields of several value-added compounds, such as recombinant proteins, gamma-aminobutyrate (GABA), and lycopene. When the pathway genes for these products were introduced, higher amounts of these compounds were produced, and specific product yields were significantly improved in the sugar transporter mutants.

## Materials and methods

### Strains and plasmids

The strains and plasmids used in this study are included in Table 1. *E. coli* W (KCTC 1039), provided by Korean Collection for Type Cultures (KCTC) was used as the host strain. Sugar transporter mutants were constructed by deleting PtsG (ADT74705), MalE (ADT77685), MglB (ADT75786), and GalP (ADT76576) to reduce the glucose uptake rate. Firstly, PtsG, the major glucose transporter in *E. coli*, was deleted by λ-red recombinase based homologous recombination, as previously described [22]. The strain was named ST2 (Table 1). Additionally, sugar transporters, such as MalE, MglB, GalP, were serially knocked out in ST2, which was named ST8 (*ΔptsG ΔmalE ΔmglB ΔgalP*) (Table 1). All deletions were confirmed by PCR. The oligonucleotides were synthesized from Bionics (Bionics, Seoul, Korea). The sequences of primers for gene deletions and confirmations are listed in Additional file 1: Table S1 and the plasmids used in this study are listed in Table 1.

**Table 1:** Strains and plasmid used in this study

	Description	Reference
Strain		
ST1	*Escherichia coli* W KCTC1039	Korean collection for type cultures
ST2	ST1 *ptsG*::FRT	This study
ST8	ST2 *mglB*::FRT *malE*::FRT *galP*::FRT	This study
STE1	ST1 harboring pEGFP	This study
STE2	ST2 harboring pEGFP	This study
STE8	ST8 harboring pEGFP	This study
STG1	ST1 *gabT*::FRT KanR FRT harboring pGABA	This study
STG2	ST2 *gabT*::FRT KanR FRT harboring pGABA	This study
STG8	ST8 *gabT*::FRT KanR FRT harboring pGABA	This study
STL1	ST1 harboring pACYC DXS and pCDF Idi IspA CrtEBI	This study
STL2	ST2 harboring pACYC DXS and pCDF Idi IspA CrtEBI	This study
STL8	ST8 harboring pACYC DXS and pCDF Idi IspA CrtEBI	This study
Plasmid		
pKD46	Red recombinase expression plasmid, *repA*, pSC101ori, P_BAD_, *gam*, *beta alpha*, *recA*, Amp^R^	Datsenko and Wanner [22]
707FLP	Flippase expression plasmid, pSC101ori, *repA*, cl578, FLPe, TetR	Generidge
pZA31 MCS	CmR, p15A ori, PLtetO, rrnB T1	Expressys
pEGFP	EGFP expression in pZA31 MCS	This study
pGABA	GltB and GltD from E. coli, GadB^mut^ (Glu89Gln/Δ452–466) and GadC^mut^(1–470) from *C. glutamicum* expression in pZA31 MCS	This study
pACYC DXS	P_BBa__J23100_::Dxs::T_BBa B1005_ in pACYC Duet-1	Jung et al. [69]
pCDF Idi IspA CrtEBI	P_BBa__J23100_::idi::T_BBa__B1005_, P_BBa__J23100_::ispA::T_BBa B1005_, P_BBa_J23100_::crtE-crtB-crtI::T_BBa B1005_ in pCDF Duet1	Jung et al. [69]

### Medium and cultivation

Luria–Bertani broth (LB; 5 g/L yeast extract, 10 g/L tryptone, 10 g/L NaCl) was used in all the genetic manipulation procedures. The culture medium was supplemented with 100 µg/mL of carbenicillin, 50 µg/mL of kanamycin, 34 µg/mL of chloramphenicol, and 50 µg/mL of spectinomycin. M9 minimal medium (6 g/L Na_2_HPO_4_, 3 g/L KH_2_PO_4_, 1 g/L NH_4_Cl, 0.5 g/L NaCl, 0.01% of Thiamine-HCl) with glucose and 1 mL of trace elements (2.86 g/L H_3_BO_3_, 1.81 g/L MnCl_2_·4H_2_O, 0.222  g/L ZnSO_4_·7H_2_O, 0.39  g/L Na_2_MoO_4_·2H_2_O, 0.079  g/L CuSO_4_·5H_2_O, 49.4  mg/L Co(NO_3_)_2_·6H_2_O, and 0.9  g/L FeCl_3_·6H_2_O) per liter, was used for flask cultivation. Strains were cultivated in 250 mL Erlenmeyer flasks with 25 mL of working volume, at 37 °C and 250 rpm. For the production of EGFP, GABA, and lycopene, strains were cultivated in 2X M9 medium (12 g/L Na_2_HPO_4_, 6 g/L KH_2_PO_4_, 2 g/L NH_4_Cl, 1 g/L NaCl, 0.01% of Thiamine-HCl) with 20 g/L of glucose and 1 mL of trace elements. When strains harboring two or more plasmids were cultivated, the culture medium was supplemented with half the concentration of antibiotics. All the chemical reagents were procured from Sigma-Aldrich (Sigma-Aldrich, St. Louis, MO, US), unless otherwise mentioned.

### Analytical method

The growth of strains was estimated by measuring the optical density at 600 nm (OD_600_) using a spectrometer DU730 (Beckman coulter, Brea, CA, US). For analysis of glucose and acetate, supernatant of the culture broth was harvested by centrifugation, followed by filtration using 0.22 µm pore syringe filter. A high performance liquid chromatography (HPLC) system, having refractive index detector Waters 2414 (Waters, Milford, MA, US), with holding temperature of 45 °C, was employed. SH1011 columns (Shodex, Tokyo, Japan) were used for separation and quantification of sugars, organic acid, and alcohols with temperature maintained at 75 °C. Dilute sulfuric acid (10 mM) was used for the HPLC mobile phase, with flow rate adjusted to 0.6 mL/min. The concentration of glucose and acetate was calculated via linear interpolation calibration using glucose and acetate standards.

The intensity of the EGFP fluorescence was measured by a microplate reader (Synergy H1; Biotek, Winooski, VT, US) with 100 µL of phosphate buffer saline-washed and diluted culture broth. Excitation was achieved at 479 nm and emission was detected at 520 nm. For detection of GABA, an HPLC UV detector system (YL9100 HPLC system; Younglin instrument, Seoul, Korea) was utilized. The HPLC system was equipped with an amino acid analysis column (Eclipse Amino acid analysis; Agilent Technology, Santa Clara, CA, US) and the temperature was maintained at 40 °C. Mobile phase A (40 mM Na_2_HPO_4_ with 1% phosphoric acid) and mobile phase B (40% acetonitrile, 40% methanol, 20% H_2_O) were adjusted for gradient flow, with a flow rate of 1.5 mL/min. The culture broth was filtered after centrifugation. The supernatant (5 µL) was mixed with 30 µL of ortho-phthalaldehyde (OPA) and borate (1:5) buffer for derivatization. The derivatives were injected and analyzed at 338 nm using UV detectors. To measure the quantity of lycopene, 200 µL of culture broth was harvested. The supernatant was removed after centrifugation (SMART R17; Hanil, Gimpo, Korea). About 1 mL of extraction solvent (mixture of equal volumes of methanol and acetone) was added to the pellet and mixed well. The solution was heated for 60 min at 65 °C with vigorous vortexing every 20 min, for sufficient elution of lycopene. After extraction, the cell debris were removed by centrifugation (21,000×*g*) and the supernatant was harvested. The absorbance of the supernatant was measured at 475 nm using a UV–Vis spectrometer (DU730; Beckman coulter, Brea, CA, US). For calculating yields, the intensity of EGFP, the quantities of GABA and lycopene were normalized by DCW and consumed glucose at early stationary phase. For measuring dry cell weight (DCW), 10 mL of culture broth was harvested and the supernatant was removed by centrifugation. Then cell pellet was resuspended and washed by distilled water for removal of remaining salts. The pellet was dried at 65 °C overnight and weighed.

### Transcriptome analysis

The culture broths of ST1, ST2, and ST8 were harvested in early exponential phase (OD_600_ ~ 1) (Additional file 1: Figure S1). The supernatant was removed by centrifugation and the pellet was used for RNA extraction procedures. A Trizol based RNA extraction kit (Hybrid R; GeneAll, Seoul, Korea) was used for RNA extraction. The RNA integrity number (RIN), rRNA ratio, and concentration of samples were checked by using the Agilent technologies 2100 Bioanalyzer (Agilent Technology, Santa Clara, CA, US). After satisfying the quality control criteria, samples were included for further analysis (Macrogen, Seoul, Korea). The Ribo-Zero rRNA Removal Kit and TruSeq stranded total RNA sample prep kit were used for RNA purification, following which, libraries were constructed (Macrogen, Seoul, Korea). The total RNA was sequenced by NovaSeq 6000 system (Macrogen, Seoul, Korea). For data analysis, *E. coli* W genomic DNA was used as reference (GCF_000184185.1_ASM18418v1) and the fold change between transcriptomes of ST1, ST2, and ST8 was calculated.

## ^13^C MFA experiment

2X M9 medium with [1,2-^13^C] glucose (Cambridge isotope Laboratories, Tewksbury, MA, US) was used in the ^13^C labeling experiments. One milliliter of cell broth in the early exponential phase (OD_600_ ~ 1) (Additional file 1: Figure S1) was centrifuged at 15,000×*g* for 10 min at 4 °C. The supernatant was removed, and the pellet was resuspended in 0.5 mL of distilled water. The wash process was repeated, and pellet was lyophilized in a freeze dryer (Hanil, Gimpo, Korea). Thereafter, 200 μL of 6 N HCl was added to hydrolyze the proteins at 110 °C for 24 h. Following hydrolysis, 200 μL of 6 N NaOH was added and the protein residues were separated using Amicon Ultra 0.5 mL 10 kD centrifugation filters (Millipore Corporation, Burlington, MA, US). The solution was fully dried using a vacuum dryer (Hanil, Gimpo, Korea) and stored at − 80 °C. The stored sample was resuspended in 50 μL pyridine. Further, 80 μL of N-(tert-butyldimethylsilyl)-N-methyl-trifluoroacetamide (MTBSTFA) was added to the derivatized proteinogenic amino acids and incubated at 70 °C for 50 min.

The GC–MS method was adapted for an Agilent gas chromatograph, equipped with a HP-5MS capillary column (30 m × 0.25 mm i.d. × 0.25 mm; Agilent Technology, Santa Clara, CA, US). A sample of 1 µL was injected in 1:10 split mode with an inlet temperature of 270 °C. The helium flow rate was 1 mL/min. The oven temperature of 80 °C was set for 2 min and then raised to 280 °C at 7 °C/min. Ion source temperature and electron impact ionization (EI) voltage were 230 °C and − 70 eV, respectively. Mass fragments of the proteinogenic amino acids were analyzed by the single ion monitoring (SIM) mode [23].

### Metabolic network model, flux analysis, and statistical analysis

The network model used for flux calculation was constructed based on a previous report [24], which included all major central metabolic pathways, lumped amino acid biosynthesis pathways, and a lumped biomass formation reaction and G-value parameters to determine the fraction of proteinogenic amino acids from a labeled glucose.

An elementary metabolite unit (EMU) based software for ^13^C MFA, INCA, was used [25, 26]. Metabolic fluxes were estimated by minimization of the differences between the measured mass isotopomer distributions (MIDs) of the proteinogenic amino acids and the simulated ones, using least squares regression. To find a global solution, the fluxes were estimated 10 times with random initial values and then a χ^2^-statistical test for goodness of fit was performed. The 95% confidence intervals for all estimated fluxes were computed using the sensitivity of the minimized variance-weighted sum of squared residuals to flux variations, using a built-in function of INCA [26, 27]. Standard deviations of fluxes were determined based on previous reports [28, 29].

## Results and discussion

### Phenotype characteristics of mutation in sugar transporters

Many microorganisms adopt the phosphotransferase system (PTS) for efficient and rapid uptake of glucose. When glucose is transported to the intracellular space through PTS, PtsG (glucose-specific EIICB component) catalyzes the phosphorylation of incoming glucose with its translocation across the cell membrane. In the absence of *ptsG*, other sugar transporters that have broad specificity, such as Mgl (methyl-galactoside transport system), Mal (maltose/maltodextrin transport system), and Gal (galactose: H + symporter) play a major role as glucose transporters [17]. In order to hinder glucose uptake, major glucose uptake pathway genes were deleted (ST2: *ΔptsG*, ST8: *ΔptsG ΔmglB ΔmalE ΔgalP*) in this study.

**Fig. 1:**
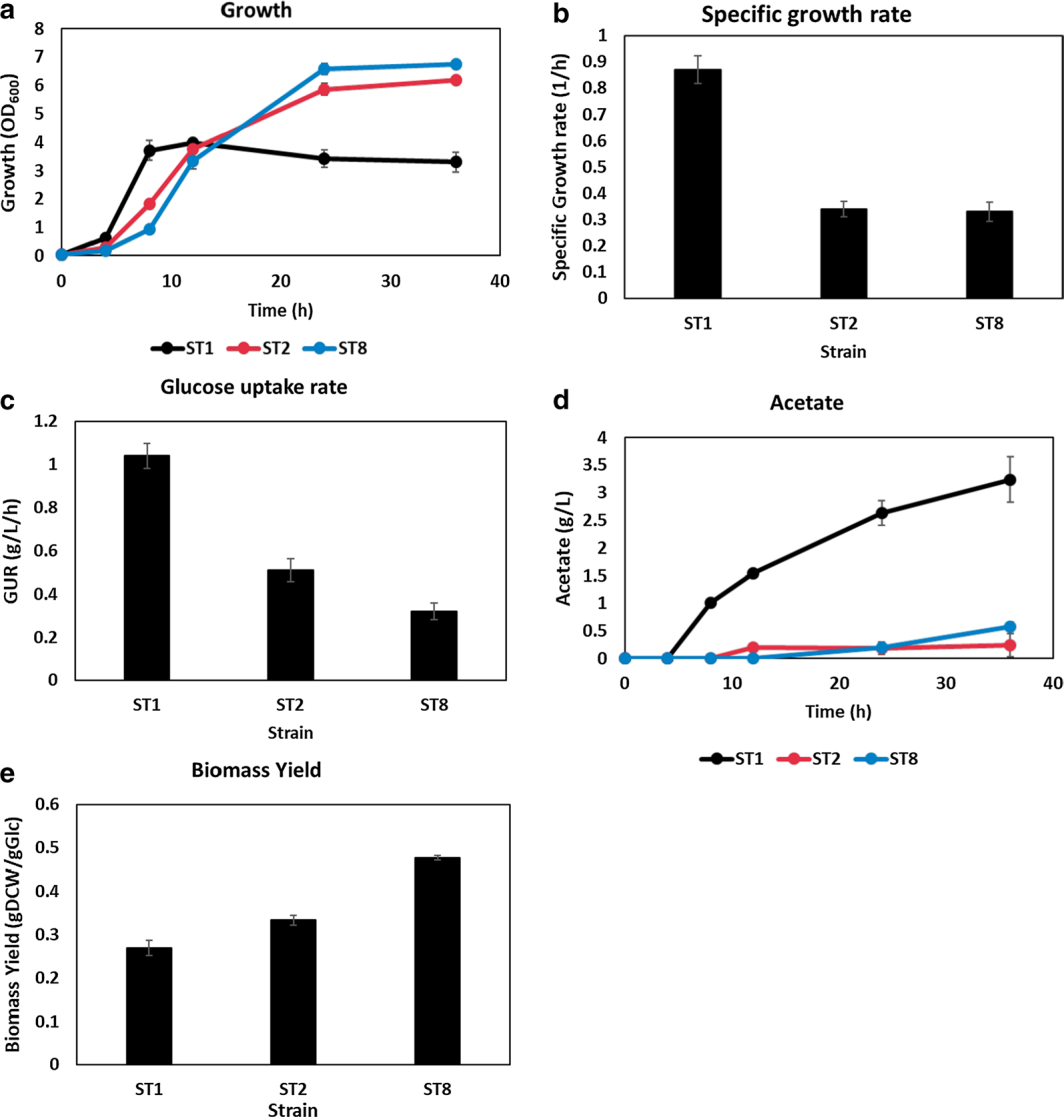
Wild type (ST1), *ptsG* mutant (ST2), *ptsG mglB malE galP* mutant (ST8) were cultivated in flasks containing M9 medium. (**a**) The growth profile and (**d**) acetate production of ST1, ST2, and ST8 is presented. **b** Specific growth rate of strains was measured by monitoring the cell growth every 30 min in exponential phase. **c** Glucose uptake rates of ST1, ST2, and ST8 were measured in exponential phase. **e** Biomass yield of strains was calculated after 12 h cultivation

*Escherichia coli* W wild type (ST1) and mutant strains (ST2 and ST8) were cultivated in shake flasks under aerobic conditions. Wild type strains reached stationary phase with maximum OD_600_ of 3.7 at 8 h. However, the sugar transporter mutants exhibited a longer lag phase and the exponential phase continued for about 24 h. The maximal OD_600_ increased about 56% and 77% in ST2 and ST8, respectively, compared to ST1 (Fig. 1a). The specific growth rate of ST1 was 0.87/h while ST2 and ST8 featured 61% lower specific growth rate (μ_ST2_: 0.34/h, μ_ST8_: 0.33/h) (Fig. 1b, Additional file 1: Figure S1). Glucose uptake rate of ST2 and ST8 were reduced by 41% and 69%, respectively, compared to that of ST1 in the early exponential phase (Fig. 1c). Acetate overflow was relieved. ST1 produced 3.2 g/L of acetate during 24 h cultivation, however, ST2 and ST8 produced much little amount of acetate during 36 h cultivation (0.24 g/L and 0.57 g/L of acetate, respectively) (Fig. 1d). According to the acetate overflow model based on FBA, acetate is produced at a specific growth rate above 0.7–0.8 [3, 30]. Therefore, it is reasonable that acetate overflow appeared in ST1 but not in ST2 and ST8. With a decrease in the production of acetate, a major by-product, biomass yield was increased. The biomass yield of ST2 and ST8 increased by 24% and 77%, respectively, compared to ST1 after 12 h cultivation (Fig. 1e). This observation accorded closely with previous results in that, improved biomass yield and decreased acetate formation appeared at low glucose uptake rate and growth rate [17, 31]. The strains with decreased glucose uptake capacity (i.e. sugar transporter mutants) sensed a glucose starved condition, although they were actually in a glucose rich condition. This is similar to the prior report that the mutant strains adapted to low glucose concentration (below 0.15 g/L) led to enhanced biomass yield [32]. Judging from the above results, it is speculated that slowing down the growth rate contributed to the efficient carbon metabolism of the host strain. Furthermore, mutation of PTS and other glucose transporter could influence not only glucose uptake capacity but also global metabolic network. Therefore, it is necessary to investigate which perturbations are connected to the changes in cell physiology.

### Transcriptome analysis of sugar transporter mutants

**Fig. 2:**
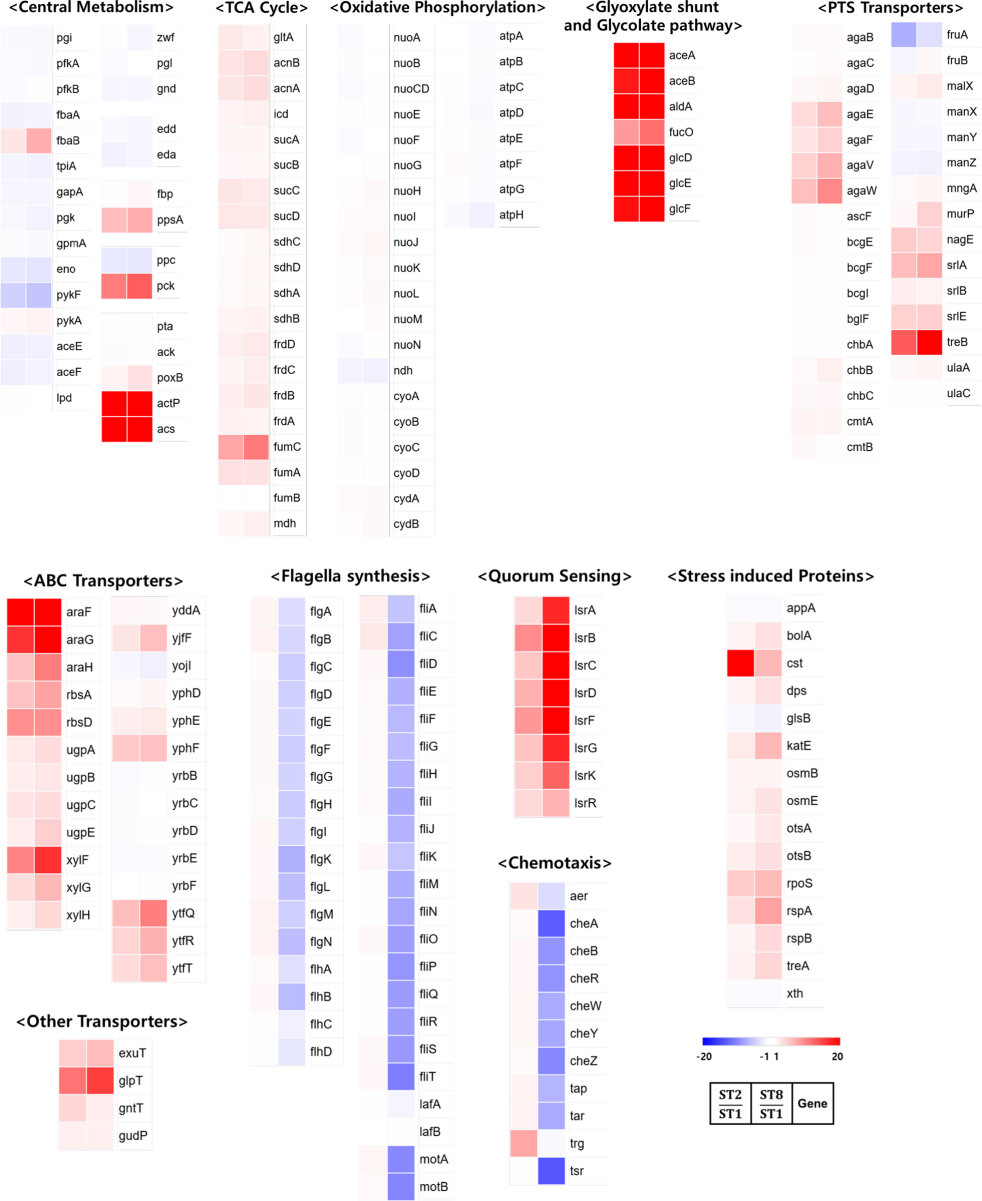
Transcriptome analysis of wild type (ST1) and sugar transporter mutants (ST2 and ST8). The first column indicates expression ratio of ST2/ST1 and the second column indicates expression ratio of ST8/ST1. The fold change (FC) between 0 to 1 was converted to − 1/FC for easy visualization of data value. The data are classified as central metabolism, TCA cycle, oxidative respiration, glyoxylate shunt, glycolate pathway, PTS transporter, ABC transporter, other transporter, flagella synthesis, quorum sensing, chemotaxis, and stress induced protein. The colors of the heat map exhibit fold change of the transcriptome in ST2 and ST8 compared to that of ST1, with maximum 20 folds to minimum − 20 folds

Various phenotypic changes were accompanied by the deletions in the sugar transporters, The deletions had a strong impact on phenotype as PTS controls the generation of intracellular cAMP by sensing the presence of glucose. Moreover, catabolite repressor/activator (Cra) plays a crucial role as a repressor or activator, in response to the intracellular concentration of fructose 1,6 bisphosphate (F1, 6BP), which affects global gene expression. Transcriptome of wild type and mutant strains were studied to examine alterations in the gene expression profile. Among the 5025 genes from the three samples (wild type ST1, sugar transporter mutants ST2 and ST8), 341 genes, which had an Reads Per Kilobase Million (RPKM) of zero, were excluded and 4684 genes were analyzed. Gene families that displayed expression-fold-change above two were considered as significant results, which accounted for 28% of the total transcriptome (1317 genes). The genes representing significant gene groups, based on their functions, were classified into four groups for discussion: 1) Central carbon metabolism and respiration (84 genes), 2) alternative transporters (62 genes), 3) quorum sensing, chemotaxis, flagella synthesis (58 genes), 4) stress-induced response (15 genes). The fold change value of the transcriptome is displayed in Fig. 2. The detailed values are tabulated (Additional file 1: Table S2).

#### Central carbon metabolism and respiration

When the glucose uptake rate was reduced, the expression of several glycolysis genes was downregulated, while the expression of gluconeogenesis genes was activated. Notably, the expression of *eno*, *pykF*, *aceE*, and *aceF*, which are enzymes involved in lower glycolysis, were downregulated in both ST2 and ST8. The expression of *fbaB* and *ppsA*, major enzymes involved in gluconeogenesis, increased remarkably. These results accorded with those of a previous study that Cra represses transcription of *eno*, *pykF*, *aceE*, and *aceF*, but activates that of *fbaB* and *ppsA* [33–35]. However, little change was observed in the transcription of genes related to upper glycolysis, pentose phosphate pathway, and ED pathway in the mutants compared to the wild type strain.

The main acetate generation pathway genes (*pta* and *ack*) were slightly downregulated but acetate transporter (*actP*) and acetyl-CoA synthase (*acs*) were strongly activated in the mutant strains. The strong induction of *acs* in *ptsG* mutants was previously reported [36]. The *actP* and *acs* genes exist in the same operon and are transcriptionally activated by CRP [37]. This suggests that acetate production is diminished significantly in the mutants. Transcription of all genes of the TCA cycle (*gltA*, *acnAB*, *icd*, *sucABCD*, *sdhAB*, *frdABCD*, *fumABC*, and *mdh*) was significantly activated. Given that most of the genes related to the TCA cycle are activated by CRP and Cra, this result corroborates well with the results of preceding studies [38, 39]. Downregulation of PEP carboxylase (*ppc*) and upregulation of PEP carboxykinase (*pck*) were also in accordance with previous reports that transcription of *ppc* is repressed by Cra, and that of *pck* is activated by Cra and CRP [39, 40]. Interestingly, *aceAB* that is activated by Cra and repressed by CRP simultaneously was highly upregulated in the sugar transporter mutants, Kim et al. defined genes regulated in opposite manner by Cra and CRP, as the “antagonization group” (*aceAB*, *pgk*, *fbaA*, *gapA*, *aceEF*), which comply with the action of Cra rather than that of CRP [41]. Unexpectedly, no significant changes were found in the expression of genes related to respiration (*nuoABCDEFGHIJKLMN*, *ndh*, *cyoABCCD*, *cydAB*, *atpABCDEFGH*), which are known to be mainly controlled in oxygenic conditions, through ArcAB and Fnr [42]. It is also reported that the transcription of *nuo* operon was enhanced by activation of Cra regulators [41]. However, these effects were not observed in the sugar transporter mutants. Overall, it was confirmed that Cra and cAMP-CRP exerted prominent effects on the transcription of key genes related to central metabolism in the sugar transporter mutants.

#### Alternative transporters

Mutations in major sugar transporter genes led to delayed glucose consumption and extended lag phase. Although most of the crucial glucose transporters were eliminated, the mutant strains still consumed glucose. It is thought that alternative sugar uptake pathways remained functional or were activated in the mutant strains. Several PTS such as *treB* (PTS for trehalose), *srlABE* (PTS for glucitol/sorbitol), *agaEFVW* (PTS for mannose/fructose/sorbose/*N*-acetylgalatosamine), *nagE* (PTS for N-acetylglucosamine), and *murP* (PTS for *N*-acetylmuramic acid) were upregulated in the sugar transporter mutants. This is consistent with prior reports that transcription of *srlABE*, *nagE*, *murP,* and *agaEFVW* are triggered by CRP [43–46]. It is well established that glucose can be imported by mannose and N-acetyl glucosamine PTS [15]. If the specificity of the uptake subunits is relaxed, glucose can be transported by other PTS because the action of phosphorous transfer from PEP is shared by a common subunit, PtsHI. However, transcription of *fruAB* (PTS for fructose) was downregulated in the mutants, which is thought to be inhibited by increase in Cra in the sugar transporter mutants [39]. In addition, various ABC transporters, *araFGH* (Arabinose ABC transporter), *glpT* (glycerol-3-phosphate transporter), *rbsA* (ribose ABC transporter), *xylFGH* (xylose ABC transporter), *gntT* (gluconate transporter), *exuT* (hexuronate transporter), *yjfF*, *yphEF,* and *ytfQRT*, were upregulated in the mutants. However, additional studies are needed to determine whether any of these activated transporters are actually involved in glucose consumption.

#### Quorum sensing, chemotaxis, flagella synthesis

*Escherichia coli* generates autoinducer-2 (AI-2) as signaling molecules, which interact with several regulators and modulate gene expression, affecting virulence, chemotaxis, and flagella synthesis [47, 48]. It has been documented that the cAMP-CRP complex regulates quorum sensing of several bacterial species such as *E. coli*, *Salmonella enterica*, and *Vibrio cholerae* [49]. Consistently, the transcription of AI-2 permease (LsrABCD), AI-2 kinase (LsrK), and the AI-2 degrading enzymes (LsrG) were activated in the sugar transporter mutants.

Interestingly, transcription of genes related to chemotaxis and flagella synthesis were slightly upregulated in ST2, but significantly downregulated in ST8. When some ligands such as nutrients or metal ions bind to transmembrane receptor proteins connected to histidine kinase (CheA) via a scaffolding protein (CheW), CheA-CheW complex phosphorylates two response regulators (CheB, CheY). Phosphorylated CheB and CheY modulate the methylation enzyme, CheR, and flagella motors, respectively [50]. As the major signal transducers in chemotaxis (CheB, CheY) were downregulated, many chemotaxis-related genes (*aer*, *cheA*, *cheB*, *cheR*, *cheW*, *cheY*, *cheZ*, *tap*, *tar*, *trg*, and *tsr*) was downregulated in ST8. In addition, master regulator for flagella synthesis, FlhDC, was downregulated in ST8. The reduced chemotaxis activity in sugar transporter mutants was confirmed by cultivation in a semi-solid medium (Additional file 1: Figure S2). Bacterial motility is powered by the proton motive force. In addition, about 8% of proteins are allocated to synthesize flagellin proteins, and 2% of total energy is consumed to synthesize and operate the flagella under normal conditions [51]. Considering that substantial energy consumption on flagella synthesis and operation, it is speculated that the cellular ATP and carbon flux was conserved, which could increase biomass yield in the sugar transporter mutants.

Although, it is reported that FlhDC can be activated by CRP, its transcription is also affected by various transcription factors [52]. For example, the expression of *flhDC* operon was repressed by ppGpp and DksA and ppGpp overrode the activation effect of CRP in poor nutrient conditions, after starvation [53]. Moreover, it was demonstrated that the intracellular concentration of ppGpp increases as growth rate decreases [54]. Therefore, it can be hypothesized that metabolic alteration by ppGpp plays a more significant role in sugar transporter mutants than in wild types. Likewise, the effect of repressors, other than CRP, is thought to be more influential to flagella synthesis in the sugar transporter mutants.

#### Stress-induced response

Sugar transporter mutants exhibited extended lag phase, and hardly consumed glucose until early exponential phase. Generally, cells in nutrient starvation activate expression of carbon starvation protein A (Cst) and post-exponential (Pex) proteins. Cst is stimulated in carbon starvation and *pex* genes are activated by carbon, nitrogen, and phosphorus starvation [55]. It was confirmed that the expression of *cst* was increased in the sugar transporter mutants in this study. Furthermore, it is reported that expression of stress response genes is regulated by *rpoS* and its proteolysis is reduced under conditions of carbon starvation [56]. Consequently, RpoS stimulates various stress induced proteins under carbon starvation. We observed that the genes for osmoprotection (*otsA*, *otsB*, *osmB*, *osmE*, and *treA*), cell morphology (*bolA*), and general stress resistance (*katE, dps*), which are regulated by RpoS, were upregulated, especially, in ST8. This denotes that the mutant strain senses itself to be under starvation conditions, despite the presence of enough glucose in the surroundings.

Bifunctional dehydratase, RspAB, was upregulated in the sugar transporter mutants. It has been demonstrated that RspAB induces degradation of homoserine lactone (HSL), which affects the expression of RpoS [57]. *E. coli,* harboring *rspAB* expressing vector, featured reduced acetate production and increased recombinant protein yield [58]. Similar cell physiology was observed in the ST8 strain, in which transcription of *rspAB* was activated, but that of *rpoS* was not affected. These results are not consistent with those of a previous report [58]. However, some other factors, such as elevated level of AI-2 signaling pathway in ST8 strain, could involve in the transcriptional upregulation of RspAB, because it was reported that AI-2 is related to osmotic stress and RpoS regulations [59, 60]. Further analysis is required to disclose the molecular function of *rspAB*. Overall, mutations in major glucose transporters caused the microorganisms to sense glucose starvation conditions, which activated stress response, mediated by carbon starvation proteins and stationary phase induced sigma factor (RpoS).

### Metabolic flux distribution

**Fig. 3:**
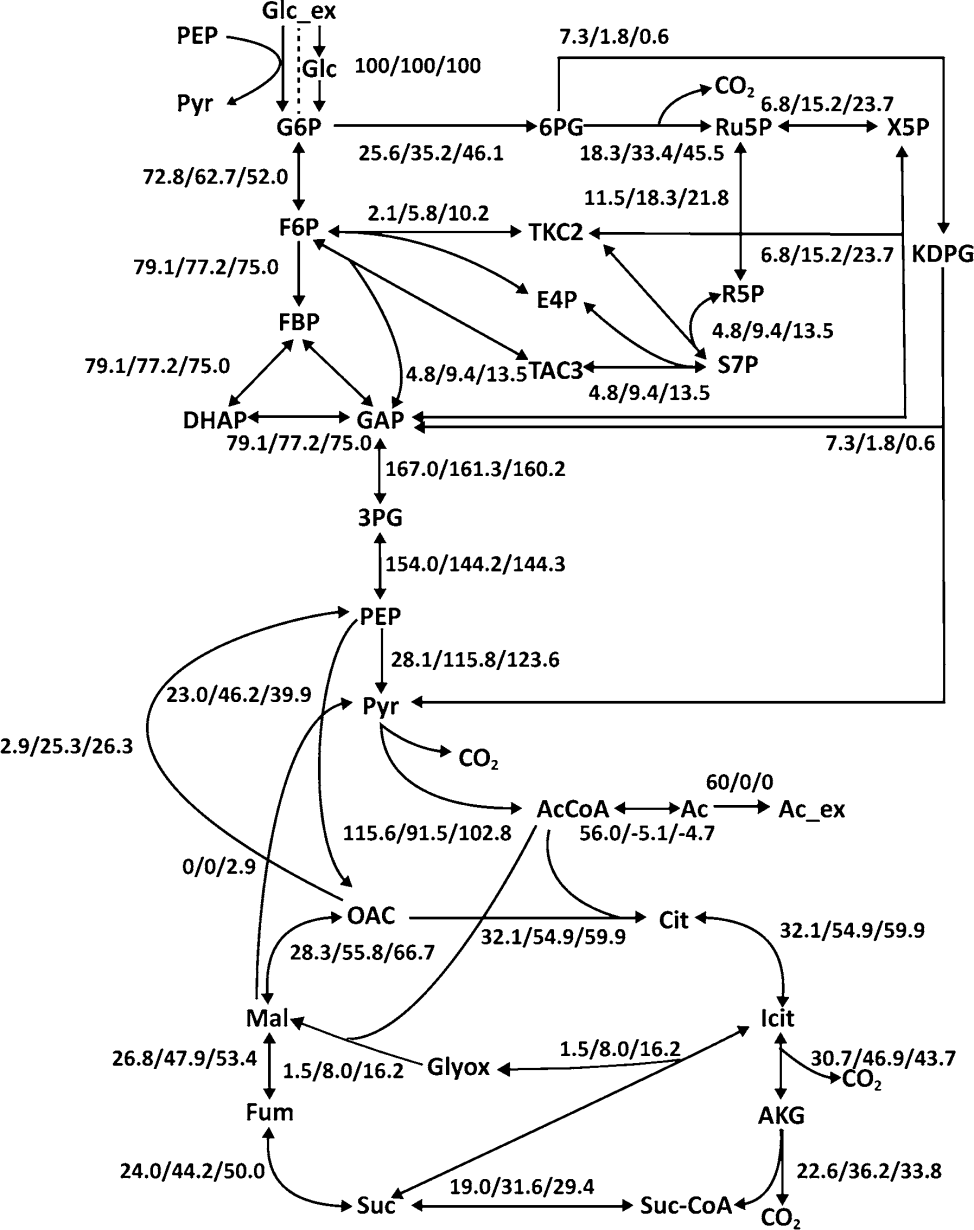
Quantification of central metabolic fluxes measured by ^13^C MFA. The three numbers separated by dashes represent the corresponding fluxes in ST1 (first), ST2 (second), and ST8 (third), respectively. PTS mediated glucose uptake reaction was removed from the networks of ST2 and ST8

To investigate perturbations in central carbon metabolism by interrupting glucose uptake, metabolic flux distributions of central carbon reactions (Fig. 3; Additional file 1: Table S5) were determined based on production rate of acetic acid and measured MIDs of proteinogenic amino acids (Additional file 1: Table S4). All ^13^C MFA results of the 3 strains showed statistically acceptable sum of squared residuals (SSR) values (Additional file 1: Table S5) and varying metabolic flux perturbations. Additionally, the contributions of individual pathways to generate key cofactors, such as NADH, FADH_2_, NADPH, and ATP, were calculated (Additional file 1: Figure S3) [20, 29, 61].

We hypothesized that ST1 uptakes glucose mainly through PTS and mutants (ST2, ST8) uptake glucose via non-specific ABC transporters. Glucose uptake through PTS consumes one molecule of PEP, however, that through ABC transporter in mutant requires additional phosphorylation step to introduce glucose to glycolysis metabolism. Therefore, The ATP generation through glycolysis decreased in sugar transporter mutant compared to control strain (Additional file 1: Figure S3). Results of ^13^C MFA showed decreased glycolysis flux ratio represented by glucose-6-phosphate isomerase (Pgi) from 72.8% to 52.0% in the mutants, while the flux ratio of the pentose phosphate and ED pathways, represented by glucose-6-phosphate dehydrogenase (Zwf), was increased [62, 63]. The higher pentose phosphate pathway flux in mutant strains clearly contributed to higher biomass yield by increasing NADPH production. The contribution of NADPH generation by transhydrogenase continuously decreased from 41% to 13% buffering the NADPH perturbations (Additional file 1: Figure S1) [64]. Moreover, the flux ratio in citrate synthase, the first step of TCA cycle, was significantly increased in ST2 and ST8. Interestingly, a significant portion of the TCA cycle flux was directed to the glyoxylate shunt pathway, which was activated from 1.5% (ST1) to 8.0% and 16.2% in ST2 and ST8, respectively. As a result, the mutant strains showed slightly increased TCA cycle-dependent NADH/FADH_2_ generation (4.2% in ST2 and 6.8% in ST8) compared with the control strain (Additional file 1: Figure S3), although all the TCA cycle genes were highly upregulated in the mutants according to the transcriptomics data. Further, ATP formation ratio via oxidative phosphorylation was moderately enhanced in ST2 and ST8 by 15.5% and 16.7%, respectively (Additional file 1: Figure S3). ST1 synthesized ATP by converting acetyl-CoA to acetate, while the mutant strains used ATP for assimilating acetate. From transcriptomics data, transcription level of *acs* was highly increased in mutant strains and the corresponding flux ratio was 56% in ST1, but -5.1% in ST2 and − 4.7% ST8, respectively. It is speculated that the metabolic perturbation in the sugar transporter mutants resulted not only in increased ATP generation from the electron transfer chain, but also in carbon conservation through the glyoxylate shunt pathway.

### Application of the mutants for metabolite production (EGFP, GABA, and lycopene)

The sugar transporter mutants exhibited: (1) improved biomass yield and reduced acetate formation, (2) strengthened TCA cycle and gluconeogenesis, (3) improved ATP conservation by losing motility. These characteristics of the sugar transporter mutants are likely to be helpful in the production of several value-added compounds.

**Fig. 4:**
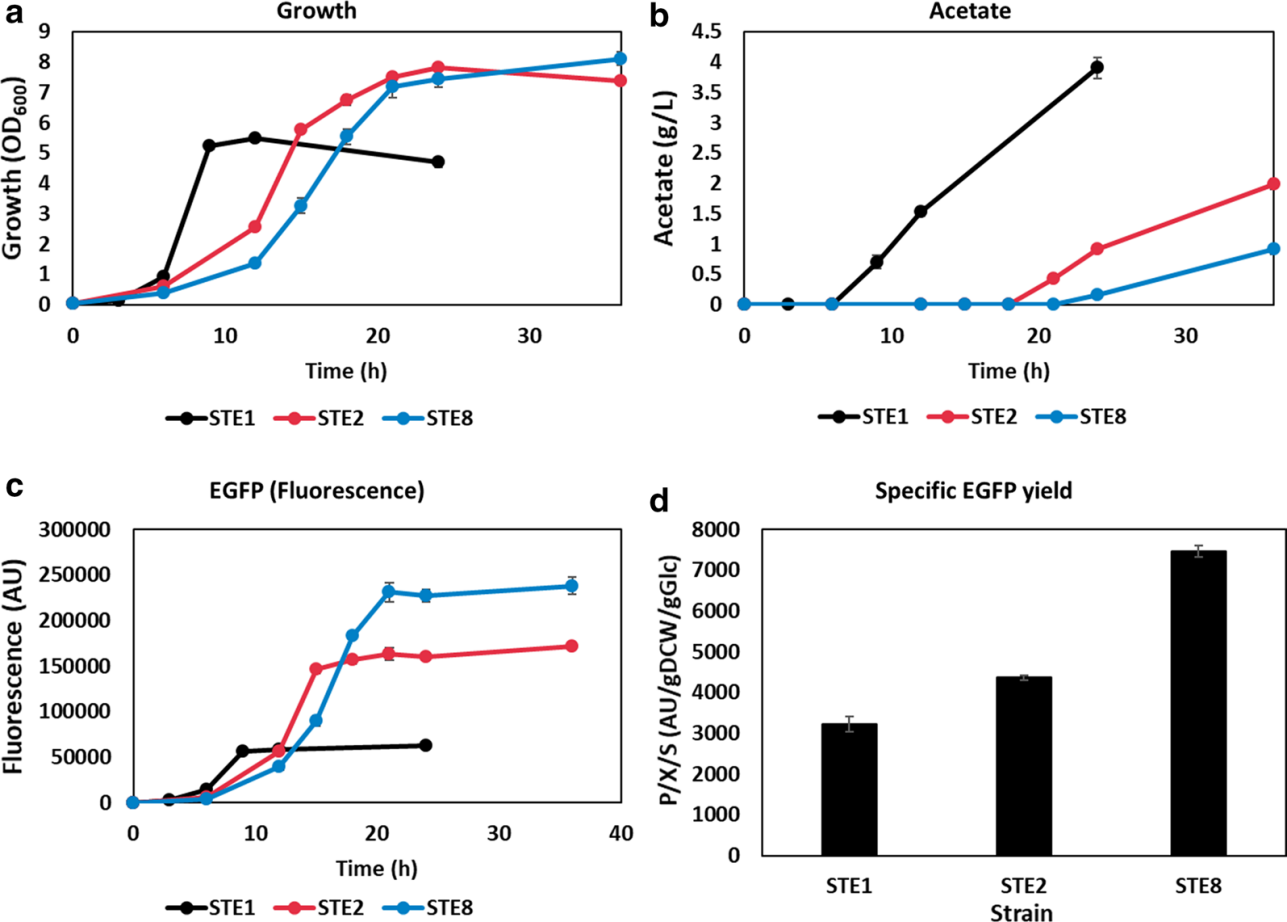
EGFP expression plasmid was introduced into ST1, ST2, and ST8 (STE1, STE2, STE8, respectively). The strains were cultivated in flasks containing 2X M9 medium. **a** The growth profile and (**b**) acetate production of STE1, STE2, and STE8 are presented. **c** The fluorescence intensity of strains monitored by microplate reader (excitation: 479 nm, emission: 520 nm). Data of STE1, STE2, and STE8 are represented by black, red, and blue lines, respectively. **d** The specific yield of EGFP was measured in the early stationary phase of cultivation. The samples of STE1, STE2, and STE8 were taken at 12 h, 24 h, and 24 h, respectively to calculate specific yield of EGFP. All data are averaged through three independent experiments

First, we attempted to take advantage of these effects in the production of recombinant proteins because the host strain produced less acetate but yielded enough amino acids and ATP. Several research groups have successfully reduced acetate production, with a subsequent increase in the production of recombinant proteins, such as DNA vaccines and glutamate dehydrogenase, in *ptsG* mutants [31, 65]. The plasmid containing the EGFP encoding gene under a constitutive promoter, was introduced into ST1, ST2, and ST8 and the resulting strains were named as STE1, STE2, and STE8, respectively. A retardation in growth rate was observed in STE2 and STE8, but eventually 35% higher maximum cell mass (OD_600_) compared to STE1, was achieved in both strains (Fig. 4a). STE2 and STE8 produced 49% and 77% lower acetate, respectively, at the end of cultivation (Fig. 4b). To quantify the intracellular EGFP expression, fluorescence intensity was measured. The linearity between the intensity of fluorescence intensity and fluorescent protein concentration has been demonstrated in previous report [66]. Interestingly, fluorescence intensity was enhanced about 160% and 282% in STE2 and STE8, respectively (Fig. 4c), resulting in a corresponding increase of 35% and 132% in the specific yields of EGFP, when calculated in the early stationary phase (Fig. 4d).

**Fig. 5:**
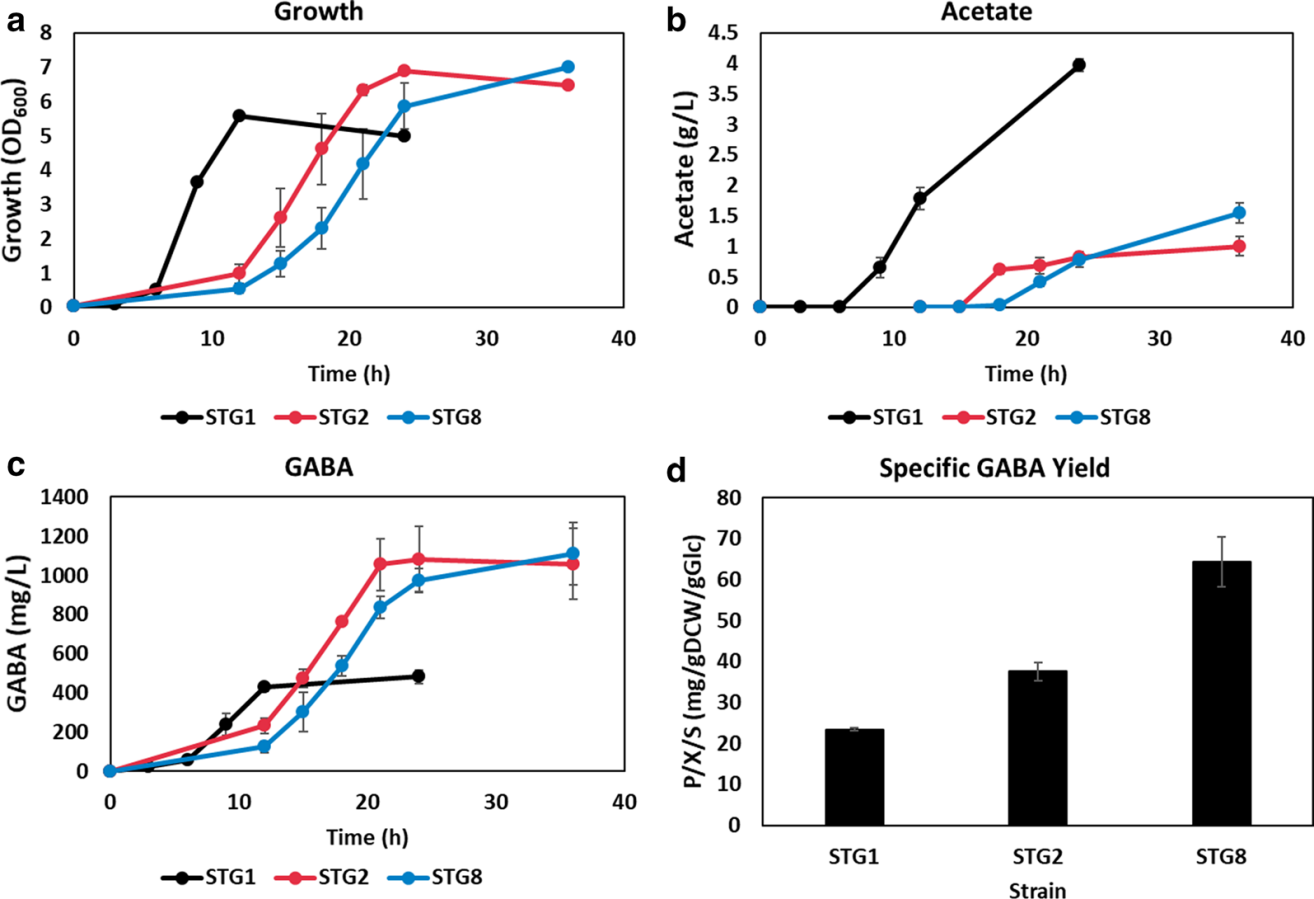
Pathway expression plasmid for GABA production was introduced into ST1, ST2, and ST8 (STG1, STG2, STG8, respectively). The strains were cultivated in flasks containing 2X M9 medium. **a** The growth profile, **b** acetate production, and **c** GABA production of STG1, STG2, and STG8 were monitored. Data of STG1, STG2, and STG8 are represented by black, red and blue lines, respectively. **d** The specific yield of GABA was calculated in the early stationary phase of cultivation. The samples of STG1, STG2, and STG8 were taken at 12 h, 24 h, and 24 h, respectively to calculate specific yield of GABA. All data are averaged through three independent experiments

Higher TCA cycle fluxes in the sugar transporter mutants are thought to be beneficial for the production of TCA cycle derived products. Indeed, improved production of succinate was reported when the glucose PTS component was mutated [67]. We attempted the same in our sugar transporter mutants during GABA production under aerobic conditions. GABA is derived from α-ketoglutarate, an intermediate of the TCA cycle, through a two-step enzyme reaction. Plasmids, *GadB*^*mut*^ (Glu89Gln/Δ452–466) and *GadC*^*mut*^ (1–470), were constructed for the expression of glutamate synthases from *Corynebacterium glutamicum* ATCC 13032, and introduced into ST1, ST2, and ST8. Furthermore, *gabT* was deleted to prevent degradation of GABA to succinate semialdehyde, and the resulting strains were named as STG1, STG2, and STG8, respectively. STG2 and STG8 exhibited retarded growth but improved maximum biomass production, compared to STG1 (Fig. 5a). Predictably, acetate production was decreased by about 75% and 61% in STG2 and STG8, respectively (Fig. 5b). Final GABA titers of STG2 and STG8 were 119% and 130% higher than that of STG1 (Fig. 5c), with improved specific GABA yields by 61% and 176%, respectively (Fig. 5d).

**Fig. 6:**
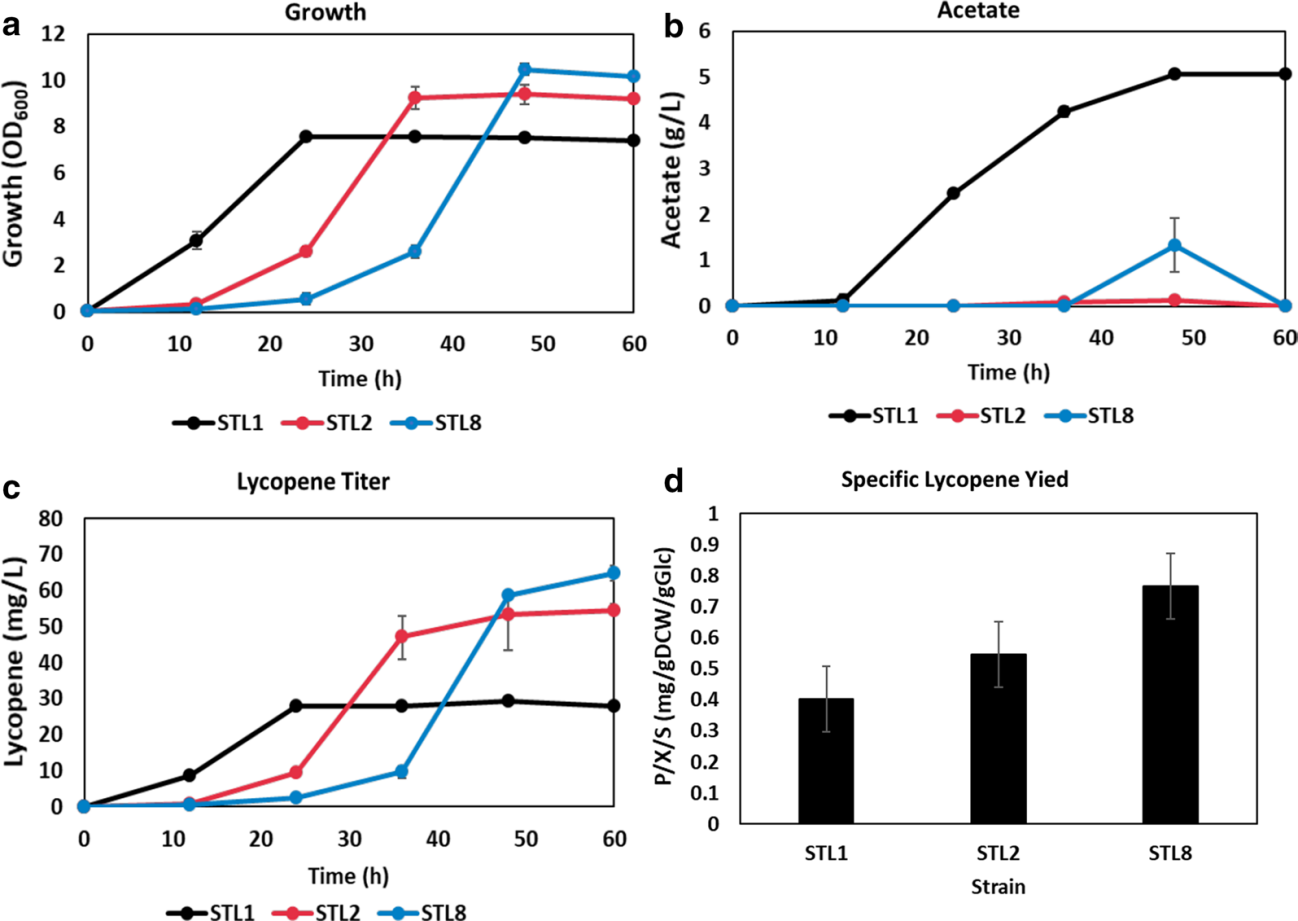
Plasmid with the lycopene pathway was introduced into ST1, ST2, and ST8 (STL1, STL2, STL8, respectively). The strains were cultivated in flasks containing 2X M9 medium. **a** The growth profile, **b** acetate production, and **c** lycopene production of STL1, STL2, and STL8 are presented. Data of STL1, STL2, and STL8 are represented by black, red, and blue lines, respectively. **d** The specific yield of lycopene was calculated in the early stationary phase of cultivation. The samples of STL1, STL2, and STL8 were taken at 24 h, 36 h, and 48 h, respectively to calculate specific yield of lycopene. All data are averaged through three independent experiments

Further, lycopene producing strains were constructed. It has been documented that *ptsG* mutants showed higher production of lycopene compared to the parental strain, however, the mechanism was not suggested in a prior study [68]. Another study demonstrated that activation of *ppsA* and repression of *gapA* were attempted to balance intracellular G3P and pyruvate, precursors of MEP pathway [69]. According to our transcriptome analysis, transcription of *gapA* was downregulated and that of *ppsA* was upregulated in the sugar transporter mutants. Lycopene pathway harboring plasmids were introduced into ST1, ST2, and ST8, which were named as STL1, STL2, and STL8, respectively. Extended lag phase was observed in STL2 and STL8 compared to that in STL1 (Fig. 6a). STL1 produced 5 g/L of acetate at the end of cultivation, however, STL2 and STL8 produced no acetate at all (Fig. 6b). The final lycopene titers were 96% and 132% higher in STL2 and STL8 (Fig. 6c), respectively. The specific lycopene yield of STL2 and STL8 was improved about 35% and 90%, respectively, as compared to that of STL1, in the stationary phase (Fig. 6d). It maybe envisaged that not only the enhanced biomass yield, but also the increased PPP flux, probably contributed to NADPH supply for lycopene production in the sugar transporter mutants.

In this study, reduced glucose uptake rate for increased efficacy of cellular metabolism was investigated. The optimization of carbon source uptake rate was indeed beneficial to efficient growth and metabolite production. However, lower glucose uptake featured extended lag phase of strains, which can cause low productivity problems. Therefore, fine-tuning of substrate uptake rate would be required for establishing speed and efficiency of metabolism of industrial strains. Recent advances in the methods for selection of slow-growing microbes using CRISPRi technology [70] and laboratory evolution, have been applied to rebalance cellular metabolism [71] and could be solutions for development of industrial strains optimized for metabolite production.

## Conclusions

Bacterial growth is closely related to the uptake of carbon sources. Although investigations involving sugar transporter mutants have been carried out in the past, not many studies exist on the physiology of the mutants. The development of omics technology allows us better understanding of intracellular events. Through transcriptome analysis, we found that global gene expression in the sugar transporter mutants was mainly regulated via cAMP-CRP and Cra. Transcription of alternative sugar transporters was upregulated, while chemotaxis response and motility were downregulated, which resulted in the conservation of cellular ATP. Furthermore, stress induced responses also affected the modulation of cellular metabolism. Changes in the intracellular metabolic flux were validated through ^13^C MFA. Through comprehensive analysis, we concluded that sugar transporter mutants can be an excellent chassis for production of several value-added compounds related to biomass and TCA cycle derivatives. Most of the high throughput enrichment in metabolic engineering for metabolites production is based on the screening of superior growth. However, our results showed the advantage of slow metabolism on bacterial growth and metabolites production. This research can be a good example of systematic approach for practical metabolic engineering.

## Supplementary information

**Additional file 1: Table S1**. Oligomers used in this study. **Table S2**. Transcriptome data for ST2, ST8 VS ST1. **Table S3**. Metabolic network model of *E. coli* used for ^13^C-MFA. **Table S4**. Mass isotopomer distributions from *E. coli* grown in batch cultures with ^12–13^C-labeled glucose. **Table S5**. Results of ^13^C-metabolic flux analysis for *E. coli* grown in batch cultures with ^12–13^C-labeled glucose. **Figure S1.** The growth curve of ST1, ST2, and ST8 in exponential phase. **Figure S2**. Motility of ST1, ST2 and ST8 in M9 semi-solid agar. **Figure S3**. Normalized cofactor balance based on ^13^C-MFA results.

## Data Availability

All data generated or analyzed during this study are included in this published article and its additional files.
