# Supplementary material for: Metabolic perturbations in mutants of glucose transporters and their applications in metabolite production in Escherichia coli
Source: Microb Cell Fact. 2019 Oct 10;18:170. doi: 10.1186/s12934-019-1224-8 (PMC6786474; doi:10.1186/s12934-019-1224-8)
Supplement: Supplementary file 1 — Additional file 1: Table S1. Oligomers used in this study. Table S2. Transcriptome data for ST2, ST8 VS ST1. Table S3. Metabolic network model of E. coli used for 13C-MFA. Table S4. Mass isotopomer distributions from E. coli grown in batch cultures with 12–13C-labeled glucose. Table S5. Results of 13C-metabolic flux analysis for E. coli grown in batch cultures with 12–13C-labeled glucose. Figure S1. The growth curve of ST1, ST2, and ST8 in exponential phase. Figure S2. Motility of ST1, ST2 and ST8 in M9 semi-solid agar. Figure S3. Normalized cofactor balance based on 13C-MFA results. [file 12934_2019_1224_MOESM1_ESM.docx]

Additional file 1.

Metabolic perturbations in mutants of glucose transporters and their applications in metabolite production in *Escherichia coli*

Hwi-Min Jung^a,1^, Dae-Kyun Im^a,1^, Jae Hyung Lim^b^, Gyoo Yeol Jung^b,c^, Min-Kyu Oh^a,*^

^a^ Department of Chemical and Biological Engineering, Korea University, 145 Anam-ro, Seongbuk-gu, Seoul 02841, Korea

^b^Department of Chemical Engineering, Pohang University of Science and Technology, 77 Cheongam-ro, Nam-gu, Pohang, Gyeongbuk 37673, Korea

^c^School of Interdisciplinary Bioscience and Bioengineering, Pohang University of Science and Technology, 77 Cheongam-ro, Nam-gu, Pohang, Gyeongbuk 37673, Korea

^1^ These two authors made equal contributions to the work described in this paper.

* Corresponding author: Min-Kyu Oh (mkoh@korea.ac.kr).

**Table S1**. Oligomers used in this study

| Name | Sequence (5` to 3`) | | Description | |  |
| --- | --- | --- | --- | --- | --- |
| ΔptsG_FKF_Fwd | AGGTCGGTAAATCGCTGATGCTGCCGGTATCCGTACTGCCTATCGCAGGT gtgtaggctggagctgcttc | | FRT-KanR-FRT cassette PCR for *ptsG* mutation | |  |
| ΔptsG_FKF_Rev | CAGTCGGGTCACCCGCCATATAACGCGGAATGTCGCCGTGGAAAACCTGA gtccatatgaatatcctcct | | FRT-KanR-FRT cassette PCR for *ptsG* mutation | |  |
| ΔptsG_C_Fwd | gtaaagttcaccgccgaaaa | | Confirmation of *ptsG* mutation | |  |
| ΔptsG_C_Rev | aatcgcctgaacaccagaac | | Confirmation of *ptsG* mutation | |  |
| ΔmglB_FKF_Fwd | CAAGATGCGAAAGCCGCGCCAGATGTTCAGCTGCTGATGAATGATTCTCA gtgtaggctggagctgcttc | | FRT-KanR-FRT cassette PCR for *mglB* mutation | |  |
| ΔmglB_FKF_Rev | AAAGGTCGCTTTCGCCTGGTTGTTAGCATCGTTCAGTACTGTGCCCGCCA gtccatatgaatatcctcct | | FRT-KanR-FRT cassette PCR for *mglB* mutation | |  |
| ΔmglB_C_Fwd | gagcttcggcgttcagtaac | | Confirmation of *mglB* mutation | |  |
| ΔmglB_C_Rev | gtggccggactttgaaatta | | Confirmation of *mglB* mutation | |  |
| ΔmalE_FKF_Fwd | ACATTATCTTCTGGGCACACGACCGCTTTGGTGGCTACGCTCAATCTGGC gtgtaggctggagctgcttc | | FRT-KanR-FRT cassette PCR for *malE* mutation | |  |
| ΔmalE_FKF_Rev | AAGCGGACATCTGCGGGATGTTCGGCATGATTTCACCTTTCTGGGCGTTT gtccatatgaatatcctcct | | FRT-KanR-FRT cassette PCR for *malE* mutation | |  |
| ΔmalE_C_Fwd | atgtgcgcatctccacatta | | Confirmation of *malE* mutation | |  |
| ΔmalE_C_Rev | acgccttatccgacaacaac | | Confirmation of *malE* mutation | |  |
| ΔgalP_FKF_Fwd | GTTCGGTGCGGCAGTCGGTGCGGTGGGCAGCGGCTGGCTCTCCTTTAAAC gtgtaggctggagctgcttc | | FRT-KanR-FRT cassette PCR for *galP* mutation | |  |
| ΔgalP_FKF_Rev | TGGCAAGTACGTTGGTCAGGCCGACAATCACGGTCCCCCACATTTGCTCG gtccatatgaatatcctcct | | FRT-KanR-FRT cassette PCR for *galP* mutation | |  |
| ΔgalP_C_Fwd | tctggcgggattactctttg | | Confirmation of *galP* mutation | |  |
| ΔgalP_C_Rev | caacgatcatgttggcaatc | | Confirmation of *galP* mutation | |  |
| ΔgabT_FKF_Fwd | ATGAGCAGCAATAAAGAGTTAATGCAGCGCCGCAGTCAGGCAGTTCCTCG gtgtaggctggagctgcttc | | FRT-KanR-FRT cassette PCR for *gabT* mutation | |  |
| ΔgabT_FKF_Rev | CTTTTTGCAGCAGATTTTCCTGCTCGAACACCTTCAACACTTCCAGCGCC gtccatatgaatatcctcct | | FRT-KanR-FRT cassette PCR for *gabT* mutation | |  |
| ΔgabT_C_Fwd | aagccaatgacaccgaattt | | Confirmation of *gabT* mutation | |  |
| ΔgabT_C_Rev | cttcgcctcagcaaaacact | | Confirmation of *gabT* mutation | |  |
| EGFP_Fwd | *ATATATGGTACC* atggtgagcaagggcgag | | EGFP ORF insertion in pZA31 MCS plasmid | |  |
| EGFP_Rev | *ATATATGTCGAC* cttctctcatccgccaaaac | | EGFP ORF insertion in pZA31 MCS plasmid | |  |
| pZA_gltB | attaaagaggagaaaggtacatgaaaccacaaggaactctac | | gltBD insertion in pZA31 MCS plasmid | |  |
| gltD_gltB | | ttagctcactgcctccatg | | *gltBD* insertion in pZA31 MCS plasmid | |
| gltB_gltD | tcatggaggcagtgagctaatg | | *gltBD* insertion in pZA31 MCS plasmid | |  |
| pZA_gltD | tatcaagcttatcgataccgctagacagccagcggcac | | *gltBD* insertion in pZA31 MCS plasmid | |  |
| pZA_gadBm | attaaagaggagaaaggtacatggataagaagcaagtaacggatttaagg | | *gadB^mut^/gadC^mut^* insertion in pZA31 MCS plasmid | |  |
| gadCm_gadBm | atccccctaagggcccgacactcagtga | | *gadB^mut^/gadC^mut^* insertion in pZA31 MCS plasmid | |  |
| gadBm_gadCm | tgtcgggcccttagggggataatatggc | | *gadB^mut^/gadC^mut^* insertion in pZA31 MCS plasmid | |  |
| pZA_gadCm | tatcaagcttatcgataccgttatttacgatcatgaacagc | | *gadB^mut^/gadC^mut^* insertion in pZA31 MCS plasmid | |  |
| pZA_gltB_gltD_gadBm_gadCm_F | gtaggacaaatccgccgccctagatccctatcagtgatagag | | *gltB/D, gadB^mut^/gadC^mut^* insertion in pZA31 MCS plasmid | |  |
| pZA_gltB_gltD_gadBm_gadCm_R | gcctatggaaaacggctttgccgcatttgtcctactcaggag | | *gltB/D, gadB^mut^/gadC^mut^* insertion in pZA31 MCS plasmid | |  |

Homology arm sequences for gene deletion are shown in capital. The annealing sequences for PCR amplification are shown in lower case. Sequences for restriction enzyme recognition are shown in italic.

**Table S2.** Transcriptome data for ST2, ST8 VS ST1. Fold change with absolute value of 2 or higher is marked in color (Mark negative in blue and positive in red). The transcriptome was classified as glycolysis, pentose phosphate pathway, acetate metabolism, anaplerotic pathway, TCA cycle, oxidative phosphorylation, glyoxylate shunt and glycolate pathway, phenylacetate degradation pathway, PTS transporters, ABC transporters, quorum sensing, chemotaxis, flagella synthesis, stress induced proteins.

| Gene_Symbol | Product | ST2/ST1 (Fold) | ST8/ST1 (Fold) |
| --- | --- | --- | --- |
| **Glycolysis** |  |  |  |
| pgi | glucose-6-phosphate isomerase | -1.43 | -1.52 |
| pfkA | ATP-dependent 6-phosphofructokinase | -1.35 | -1.48 |
| pfkB | 6-phosphofructokinase II | -1.29 | 1.22 |
| fbaA | class II fructose-bisphosphate aldolase | -1.94 | -1.85 |
| fbaB | fructose-bisphosphate aldolase | 2.92 | 7.03 |
| tpiA | triose-phosphate isomerase | -1.82 | -1.94 |
| gapA | aldehyde dehydrogenase | -1.68 | -1.69 |
| pgk | phosphoglycerate kinase | -1.52 | -1.86 |
| gpmA | 2,3-bisphosphoglycerate-dependent phosphoglycerate mutase | -1.26 | -1.42 |
| eno | enolase | -2.76 | -2.93 |
| pykF | pyruvate kinase I | -4.43 | -5.41 |
| pykA | pyruvate kinase II | 1.63 | 1.88 |
| aceE | pyruvate dehydrogenase E1 component | -2.19 | -1.99 |
| aceF | dihydrolipoyllysine-residue acetyltransferase component of pyruvate dehydrogenase complex | -2.24 | -1.93 |
| lpd | dihydrolipoyl dehydrogenase | 1.16 | 1.03 |
| **Pentose phosphate pathway** |  |  |  |
| zwf | glucose-6-phosphate 1-dehydrogenase | -1.15 | -1.68 |
| pgl | 6-phosphogluconolactonase | -1.29 | 1.02 |
| gnd | phosphogluconate dehydrogenase (NADP(+)-dependent, decarboxylating) | -1.56 | -1.58 |
| **ED pathway** |  |  |  |
| edd | phosphogluconate dehydratase | -1.43 | -1.64 |
| eda | KHG/KDPG aldolase | -1.94 | -1.78 |
| **Gluconeogenesis** |  |  |  |
| fbp | fructose 1,6-bisphosphatase | 1.15 | 1.58 |
| ppsA | phosphoenolpyruvate synthase | 5.89 | 7.11 |
| **Acetate metabolism** |  |  |  |
| pta | phosphate acetyltransferase | -1.12 | 1.10 |
| ack | acetate kinase | -1.25 | -1.05 |
| poxB | ubiquinone-dependent pyruvate dehydrogenase | 1.93 | 3.27 |
| actP | cation acetate symporter | 64.60 | 31.35 |
| acs | acetyl-coenzyme A synthetase | 36.39 | 41.00 |
| **Anaplerotic pathway** |  |  |  |
| ppc | phosphoenolpyruvate carboxylase | -2.64 | -2.81 |
| pck | phosphoenolpyruvate carboxykinase (ATP) | 10.70 | 12.96 |
| **TCA cycle** |  |  |  |
| gltA | type II citrate synthase | 2.69 | 2.06 |
| acnB | bifunctional aconitate hydratase 2/2-methylisocitrate dehydratase | 2.99 | 3.72 |
| acnA | aconitate hydratase AcnA | 2.88 | 3.58 |
| icd | NADP-dependent isocitrate dehydrogenase | 1.88 | 2.12 |
| sucA | 2-oxoglutarate dehydrogenase subunit E1 | 1.77 | 1.77 |
| sucB | dihydrolipoyllysine-residue succinyltransferase | 1.56 | 1.66 |
| sucC | succinyl-CoA ligase subunit beta | 2.89 | 2.66 |
| sucD | succinyl-CoA ligase subunit alpha | 2.82 | 2.71 |
| sdhC | succinate dehydrogenase cytochrome b556 subunit | 1.30 | 1.65 |
| sdhD | succinate dehydrogenase hydrophobic membrane anchor subunit | 1.37 | 1.68 |
| sdhA | succinate dehydrogenase flavoprotein subunit | 1.47 | 1.64 |
| sdhB | succinate dehydrogenase iron-sulfur subunit | 1.83 | 2.08 |
| frdD | fumarate reductase subunit D | 2.16 | 2.61 |
| frdC | fumarate reductase subunit C | 1.76 | 2.45 |
| frdB | fumarate reductase iron-sulfur subunit | 2.25 | 2.88 |
| frdA | fumarate reductase flavoprotein subunit | 2.08 | 1.95 |
| fumC | class II fumarate hydratase | 7.63 | 10.84 |
| fumA | fumarate hydratase | 3.24 | 3.13 |
| fumB | fumarate hydratase class I, anaerobic | 1.03 | 1.00 |
| mdh | malate dehydrogenase | 1.66 | 2.12 |
| **Oxidative phosphorylation** |  |  |  |
| nuoA | NADH-quinone oxidoreductase subunit A | -1.34 | 1.03 |
| nuoB | NADH-quinone oxidoreductase subunit B | -1.25 | 1.02 |
| nuoCD | NADH-quinone oxidoreductase subunit C/D | -1.34 | 1.06 |
| nuoE | NADH-quinone oxidoreductase subunit E | -1.06 | 1.11 |
| nuoF | NADH-quinone oxidoreductase subunit NuoF | -1.30 | -1.01 |
| nuoG | NADH-quinone oxidoreductase subunit NuoG | 1.03 | 1.24 |
| nuoH | NADH-quinone oxidoreductase subunit H | 1.21 | 1.54 |
| nuoI | NADH-quinone oxidoreductase subunit I | 1.31 | 1.46 |
| nuoJ | NADH-quinone oxidoreductase subunit J | 1.36 | 1.65 |
| nuoK | NADH-quinone oxidoreductase subunit K | 1.11 | 1.48 |
| nuoL | NADH-quinone oxidoreductase subunit L | 1.13 | 1.52 |
| nuoM | NADH-quinone oxidoreductase subunit M | 1.01 | 1.27 |
| nuoN | NADH-quinone oxidoreductase subunit N | -1.28 | 1.16 |
| ndh | NADH dehydrogenase | -1.81 | -2.08 |
| cyoA | cytochrome o ubiquinol oxidase subunit II | -1.18 | 1.11 |
| cyoB | cytochrome ubiquinol oxidase subunit I | -1.23 | -1.07 |
| cyoC | cytochrome bo(3) ubiquinol oxidase subunit 3 | -1.19 | -1.09 |
| cyoD | cytochrome bo(3) ubiquinol oxidase subunit 4 | 1.05 | 1.11 |
| cydA | cytochrome bd-I ubiquinol oxidase subunit 1 | 1.37 | 1.49 |
| cydB | cytochrome bd-I ubiquinol oxidase subunit 2 | 1.17 | 1.53 |
| atpA | ATP synthase subunit alpha | -1.02 | -1.28 |
| atpB | ATP synthase subunit A | 1.02 | -1.26 |
| atpC | ATP synthase epsilon chain | -1.05 | -1.38 |
| atpD | ATP synthase subunit beta | -1.17 | -1.48 |
| atpE | ATP synthase subunit C | 1.06 | -1.25 |
| atpF | ATP synthase subunit B | 1.27 | -1.27 |
| atpG | ATP synthase subunit gamma | -1.09 | -1.38 |
| atpH | ATP synthase subunit delta | -1.46 | -2.05 |
| **Glyoxylate shunt and glycolate pathway** |  |  |  |
| aceA | isocitrate lyase | 21.95 | 39.77 |
| aceB | malate synthase A | 18.21 | 30.27 |
| aldA | aldehyde dehydrogenase | 23.22 | 31.52 |
| fucO | lactaldehyde reductase | 8.50 | 11.76 |
| glcD | glycolate oxidase | 46.74 | 72.62 |
| glcE | glycolate oxidase subunit GlcE | 28.53 | 41.40 |
| glcF | glycolate oxidase iron-sulfur subunit | 19.09 | 24.25 |

| **PTS transporter** |  |  |  |
| --- | --- | --- | --- |
| agaB | PTS N-acetylgalactosamine transporter subunit IIB | 1.30 | 1.27 |
| agaC | N-acetylgalactosamine permease IIC component 1 | -1.06 | 1.30 |
| agaD | PTS N-acetylgalactosamine transporter subunit IID | 1.49 | 1.74 |
| agaE | PTS N-acetylgalactosamine transporter subunit IID | 3.66 | 5.81 |
| agaF | PTS N-acetylgalactosamine transporter subunit IIA | 2.92 | 4.34 |
| agaV | PTS N-acetylgalactosamine transporter subunit IIB | 4.40 | 6.88 |
| agaW | PTS mannose/fructose/sorbose/N-acetylgalactosamine transporter subunit IIC | 5.79 | 9.86 |
| ascF | PTS cellobiose/arbutin/salicin transporter subunit IIBC | 1.28 | 1.19 |
| bcgE | PTS sugar transporter subunit IIB | 1.29 | 1.12 |
| bcgF | PTS sugar transporter subunit IIC | -1.04 | 1.07 |
| bcgI | PTS lactose/cellobiose transporter subunit IIA | -1.07 | -1.12 |
| bglF | PTS beta-glucoside transporter subunit IIABC | -1.06 | -1.07 |
| chbA | N,N'-diacetylchitobiose-specific phosphotransferase enzyme IIA component | 1.25 | -1.16 |
| chbB | PTS sugar transporter subunit IIB | 1.44 | 2.14 |
| chbC | N,N'-diacetylchitobiose permease IIC component | 1.53 | 1.59 |
| cmtA | PTS mannitol cryptic EIICB component | 1.92 | 1.71 |
| cmtB | mannitol-specific cryptic phosphotransferase enzyme IIA component | 1.50 | 1.18 |
| fruA | PTS fructose transporter subunit IIBC | -6.97 | -3.37 |
| fruB | bifunctional PTS fructose transporter subunit IIA/HPr protein | -1.75 | 1.51 |
| malX | PTS maltose- and glucose EIICB component | 1.93 | 2.57 |
| manX | PTS mannose transporter subunit EIIAB | -1.59 | -1.41 |
| manY | PTS mannose/fructose/sorbose transporter subunit IIC | -1.56 | -1.61 |
| manZ | PTS mannose transporter subunit IID | -1.88 | -1.94 |
| mngA | PTS 2-O-a-mannosyl-D-glycerate transporter subunit IIABC | 1.38 | 1.77 |
| murP | PTS N-acetylmuramic acid EIIBC component | 1.57 | 4.37 |
| nagE | PTS N-acetyl glucosamine transporter subunit IIABC | 4.82 | 4.16 |
| srlA | glucitol/sorbitol permease IIC component | 5.99 | 7.65 |
| srlB | glucitol/sorbitol-specific phosphotransferase enzyme IIA component | 2.40 | 1.98 |
| srlE | PTS sorbitol transporter subunit IIB | 4.38 | 4.60 |
| treB | PTS trehalose transporter subunit IIBC | 13.30 | 33.72 |
| ulaA | PTS ascorbate transporter subunit IIC | 1.51 | 1.75 |
| ulaC | ascorbate-specific phosphotransferase enzyme IIA component | 1.09 | 1.12 |
| **ABC transporter** |  |  |  |
| araF | arabinose ABC transporter substrate-binding protein | 24.77 | 39.01 |
| araG | arabinose import ATP-binding protein AraG | 16.18 | 25.63 |
| araH | arabinose ABC transporter permease | 5.58 | 10.69 |
| rbsA | ribose ABC transporter ATP-binding protein RbsA | 5.52 | 7.91 |
| rbsD | D-ribose pyranase | 9.36 | 9.36 |
| ugpA | glycerol-3-phosphate transporter permease | 2.64 | 3.50 |
| ugpB | branched chain amino acid ABC transporter substrate-binding protein | 2.26 | 2.82 |
| ugpC | glycerol-3-phosphate ABC transporter ATP-binding protein | 2.94 | 3.56 |
| ugpE | sn-glycerol 3-phosphate ABC transporter permease | 2.30 | 4.54 |
| xylF | xylose ABC transporter substrate-binding protein | 10.06 | 16.36 |
| xylG | xylose ABC transporter ATP-binding protein | 3.51 | 6.27 |
| xylH | xylose ABC transporter permease | 2.16 | 3.88 |
| yddA | ABC transporter ATP-binding protein/permease | 1.57 | 1.52 |
| yjfF | sugar ABC transporter permease YjfF | 3.00 | 5.74 |
| yojI | microcin J25 efflux ABC transporter YojI | -1.61 | -2.11 |
| yphD | ABC transporter permease | 1.29 | 1.72 |
| yphE | sugar ABC transporter ATP-binding protein | 2.25 | 2.56 |
| yphF | sugar ABC transporter substrate-binding protein | 5.06 | 5.63 |
| yrbB | phospholipid ABC transporter-binding protein | -1.29 | -1.10 |
| yrbC | phospholipid-binding protein | -1.19 | -1.03 |
| yrbD | outer membrane lipid asymmetry maintenance protein MlaD | -1.25 | -1.10 |
| yrbE | phospholipid ABC transporter permease | -1.35 | -1.30 |
| yrbF | phospholipid ABC transporter ATP-binding protein MlaF | -1.01 | -1.14 |
| ytfQ | ABC transporter substrate-binding protein | 5.81 | 10.64 |
| ytfR | sugar ABC transporter ATP-binding protein | 4.24 | 6.83 |
| ytfT | ABC transporter permease | 3.70 | 5.77 |
| **Other transporter** |  |  |  |
| exuT | hexuronate transporter | 4.61 | 5.94 |
| glpT | glycerol-3-phosphate transporter | 11.36 | 15.44 |
| gntT | gluconate transporter | 4.11 | 2.29 |
| gudP | MFS transporter | 2.18 | 2.19 |

| **Quorum sensing** |  |  |  |
| --- | --- | --- | --- |
| lsrA | autoinducer 2 ABC transporter ATP-binding protein LsrA | 3.90 | 17.16 |
| lsrB | autoinducer 2 ABC transporter substrate-binding protein | 9.54 | 33.19 |
| lsrC | autoinducer 2 ABC transporter permease LsrC | 5.22 | 22.19 |
| lsrD | autoinducer 2 import system permease LsrD | 6.79 | 24.40 |
| lsrF | 3-hydroxy-5-phosphonooxypentane-2,4-dione thiolase LsrF | 8.76 | 27.60 |
| lsrG | autoinducer 2-degrading protein LsrG | 5.50 | 17.19 |
| lsrK | autoinducer-2 kinase | 4.63 | 12.55 |
| lsrR | LsrR family transcriptional regulator | 4.00 | 6.70 |
| **Chemotaxis** |  |  |  |
| aer | PAS domain S-box protein | 3.14 | -3.60 |
| cheA | chemotaxis protein CheA | 1.30 | -13.15 |
| cheB | chemotaxis response regulator protein-glutamate methylesterase | 1.62 | -8.97 |
| cheR | chemotaxis protein methyltransferase | 1.60 | -9.05 |
| cheW | chemotaxis protein CheW | 1.70 | -7.28 |
| cheY | two-component system response regulator | 1.67 | -7.57 |
| cheZ | protein phosphatase CheZ | 1.33 | -9.90 |
| tap | methyl-accepting chemotaxis protein | 1.88 | -6.53 |
| tar | methyl-accepting chemotaxis protein II | 1.92 | -7.28 |
| trg | HAMP domain-containing protein | 7.53 | -1.41 |
| tsr | methyl-accepting chemotaxis protein | 1.04 | -13.59 |
| **Flagella synthesis** |  |  |  |
| flgA | flagellar basal body P-ring formation protein FlgA | 1.78 | -3.58 |
| flgB | flagellar biosynthesis protein FlgB | 1.94 | -3.97 |
| flgC | flagellar basal body rod protein FlgC | 1.30 | -4.76 |
| flgD | flagellar hook assembly protein FlgD | 1.55 | -4.59 |
| flgE | flagellar hook protein FlgE | 1.55 | -4.30 |
| flgF | flagellar basal body rod protein FlgF | 1.51 | -4.70 |
| flgG | flagellar basal body rod protein FlgG | 1.50 | -4.25 |
| flgH | flagellar L-ring protein | 1.37 | -4.96 |
| flgI | flagellar P-ring protein | 1.65 | -4.51 |
| flgK | flagellar hook-associated protein FlgK | 1.59 | -6.87 |
| flgL | flagellar hook-filament junction protein FlgL | 1.56 | -5.81 |
| flgM | anti-sigma-28 factor FlgM | 1.99 | -4.58 |
| flgN | flagella synthesis protein FlgN | 2.00 | -5.89 |
| flhA | flagellar biosynthesis protein FlhA | 1.43 | -3.34 |
| flhB | flagellar biosynthetic protein FlhB | 1.56 | -6.07 |
| flhC | flagellar transcriptional regulator FlhC | 1.22 | -2.15 |
| flhD | flagellar transcriptional activator FlhD | -1.17 | -2.79 |
| fliA | RNA polymerase sigma factor FliA | 2.24 | -5.41 |
| fliC | flagellin FliC | 2.78 | -7.84 |
| fliD | flagellar filament capping protein FliD | 1.61 | -9.44 |
| fliE | flagellar hook-basal body complex protein FliE | 1.46 | -6.80 |
| fliF | flagellar basal body M-ring protein FliF | 1.36 | -6.19 |
| fliG | flagellar motor switch protein FliG | 1.43 | -5.97 |
| fliH | flagellar assembly protein FliH | 1.40 | -6.64 |
| fliI | flagellum-specific ATP synthase FliI | 1.41 | -7.33 |
| fliJ | flagellar protein FliJ | 1.18 | -6.67 |
| fliK | flagellar hook length control protein FliK | 1.60 | -5.31 |
| fliM | flagellar motor switch protein FliM | 1.09 | -7.13 |
| fliN | flagellar motor switch protein FliN | 1.09 | -7.63 |
| fliO | flagellar protein FliO | 1.53 | -8.20 |
| fliP | flagellar biosynthesis protein FliP | -1.06 | -8.87 |
| fliQ | flagellar biosynthetic protein FliQ | -1.06 | -8.41 |
| fliR | flagellar type III secretion system protein FliR | -1.06 | -8.26 |
| fliS | flagella export chaperone FliS | 1.54 | -8.62 |
| fliT | flagellar protein FliT | 1.58 | -10.60 |
| lafA | lateral flagellin LafA | -1.04 | -1.77 |
| lafB | flagellar hook protein | -1.03 | -1.17 |
| motA | flagellar motor stator protein MotA | 1.65 | -9.78 |
| motB | motility protein B | 1.43 | -10.08 |

| **Stress induced protein** |  |  |  |
| --- | --- | --- | --- |
| appA | phytase AppA | -1.37 | -1.30 |
| bolA | protein BolA | 1.87 | 3.27 |
| cst | carbon starvation protein A | 22.30 | 6.31 |
| dps | DNA starvation/stationary phase protection protein | 1.90 | 3.27 |
| glsB | glutaminase 2 | -1.55 | -1.99 |
| katE | catalase HPII | 2.57 | 6.32 |
| osmB | osmotically-inducible lipoprotein B | 1.89 | 1.84 |
| osmE | osmotically-inducible lipoprotein E | 2.00 | 3.14 |
| otsA | trehalose-6-phosphate synthase | 1.56 | 2.80 |
| otsB | trehalose-6-phosphate phosphatase | 2.29 | 3.36 |
| rpoS | RNA polymerase sigma factor RpoS | 4.79 | 6.17 |
| rspA | starvation-sensing protein RspA | 3.25 | 8.04 |
| rspB | starvation-sensing protein RspB | 1.76 | 3.85 |
| treA | trehalase | 2.05 | 4.03 |
| xth | exodeoxyribonuclease III | -1.34 | -1.27 |

**Table S3.** Metabolic network model of *E.coli* used for 13C-MFA

| **Glycolysis** | | | | | |
| --- | --- | --- | --- | --- | --- |
| ST1 | Reaction | Carbon atom rearrangement | ST2/8 | Reaction | Carbon atom rearrangement |
| v1 | Glc_EX + PEP → G6P + Pyr | abcdef + ghi → abcdef + ghi | v1 | Glc_EX → Glc | abcdef → abcdef |
| v2 | G6P ↔ F6P | abcdef ↔ abcdef | v2 | Glc +ATP → G6P | abcdef → abcdef |
| v3 | F6P + ATP → FBP | abcdef → abcdef | v3 | G6P ↔ F6P | abcdef ↔ abcdef |
| v4 | FBP ↔ DHAP + GAP | abcdef ↔ cba + def | v4 | F6P + ATP → FBP | abcdef → abcdef |
| v5 | DHAP ↔ GAP | abc ↔ abc | v5 | FBP ↔ DHAP + GAP | abcdef ↔ cba + def |
| v6 | GAP ↔ 3PG + ATP + NADH | abc ↔ abc | v6 | DHAP ↔ GAP | abc ↔ abc |
| v7 | 3PG ↔ PEP | abc ↔ abc | v7 | GAP ↔ 3PG + ATP + NADH | abc ↔ abc |
| v8 | PEP → Pyr + ATP | abc → abc | v8 | 3PG ↔ PEP | abc ↔ abc |
|  |  |  | v9 | PEP → Pyr + ATP | abc → abc |
| **Pentose Phosphate Pathway** | | | | | |
| ST1(ST2/8) | | Reaction | | Carbon atom rearrangement | |
| v9(v10) | | G6P → 6PG + NADPH | | abcdef → abcdef | |
| v10(v11) | | 6PG → Ru5P + CO_2_ + NADPH | | abcdef → bcdef + a | |
| v11(v12) | | Ru5P ↔ X5P | | abcde ↔ abcde | |
| v12(v13) | | Ru5P ↔ R5P | | abcde ↔ abcde | |
| v13(v14) | | X5P ↔ TKC2 + GAP | | abcde ↔ ab + cde | |
| v14(v15) | | F6P ↔ TKC2 + E4P | | abcdef ↔ ab + cdef | |
| v15(v16) | | S7P ↔ TKC2 + R5P | | abcdefg ↔ ab+ cdefg | |
| v16(v17) | | F6P ↔ TAC3 + GAP | | abcdef ↔ abc + def | |
| v17(v18) | | S7P ↔ TAC3 + E4P | | abcdefg ↔ abc + defg | |
| **Entner-Doudoroff Pathway** | | | | | |
| ST1(ST2/8) | | Reaction | | Carbon atom rearrangement | |
| v18(v19) | | 6PG → KDPG | | abcdef → abcdef | |
| v19(v20) | | KDPG → Pyr + GAP | | abcdef → abc + def | |
| **TCA Cycle** | | | | | |
| ST1(ST2/8) | | Reaction | | Carbon atom rearrangement | |
| v20(v21) | | Pyr → AcCoA + CO_2_ + NADH | | abc → bc + a | |
| v21(v22) | | OAC + AcCoA → Cit | | abcd + ef → dcbfea | |
| v22(v23) | | Cit ↔ ICIT | | abcdef ↔ abcdef | |
| v23(v24) | | ICIT ↔ AKG + CO_2_ + NADPH | | abcdef ↔ abcde + f | |
| v24(v25) | | AKG → SucCoA + CO_2_ + NADH | | abcde → bcde + a | |
| v25(v26) | | SucCoA ↔ Suc + ATP | | abcd ↔ abcd | |
| v26(v27) | | Suc ↔ Fum + FADH2 | | abcd ↔ abcd | |
| v27(v28) | | Fum ↔ Mal | | abcd ↔ abcd | |
| v28(v29) | | Mal ↔ OAC + NADH | | abcd ↔ abcd | |
| **Glyoxylate Shunt** | | | | | |
| ST1(ST2/8) | | Reaction | | Carbon atom rearrangement | |
| v29(v30) | | ICIT → Glyox + Suc | | abcdef → ab + edcf | |
| v30(v31) | | Glyox + AcCoA → Mal | | ab + cd → abdc | |
| **Amphibolic Reactions** | | | | | |
| ST1(ST2/8) | | Reaction | | Carbon atom rearrangement | |
| v31(v32) | | Mal → Pyr + CO_2_ + NADPH | | abcd → abc + d | |
| v32(v33) | | Mal → Pyr + CO_2_ + NADH | | abcd → abc + d | |
| v33(v34) | | PEP + CO_2_ → OAC | | abc + d → abcd | |
| v34(v35) | | OAC + ATP → PEP + CO_2_ | | abcd → abc + d | |
| **Acetic Acid Formation** | | | | | |
| ST1(ST2/8) | | Reaction | | Carbon atom rearrangement | |
| v35(v36) | | AcCoA ↔ Ac + ATP | | ab ↔ ab | |
| **Amino Acid Biosynthesis** | | | | | |
| ST1(ST2/8) | | Reaction | | Carbon atom rearrangement | |
| v36(v37) | | AKG + NADPH + NH_3_ → Glu | | abcde → abcde | |
| v37(v38) | | Glu + ATP + NH_3_ → Gln | | abcde → abcde | |
| v38(v39) | | Glu + ATP + 2 NADPH → Pro | | abcde → abcde | |
| v39(v40) | | Glu + CO_2_ + Gln + Asp + AcCoA + 5 ATP + NADPH → Arg + AKG + Fum + Ac | | abcde + f + ghijk + lmno + pq → abcdef + ghijk + lmno + pq | |
| v40(v41) | | OAC + Glu → Asp + AKG | | abcd + efghi → abcd + efghi | |
| v41(v42) | | Asp + 2 ATP + NH_3_ → Asn | | abcd → abcd | |
| v42(v43) | | Pyr + Glu → Ala + AKG | | abc + defgh → abc + defgh | |
| v43(v44) | | 3PG + Glu → Ser + AKG + NADH | | abc + defgh → abc + defgh | |
| v44(v45) | | Ser ↔ Gly + MEETHF | | abc ↔ ab + c | |
| v45(v46) | | Gly ↔ CO_2_ + MEETHF + NADH + NH3 | | ab ↔ a + b | |
| v46(v47) | | Thr → Gly + AcCoA + NADH | | abcd → ab + cd | |
| v47(v48) | | Ser + AcCoA + 3 ATP + 4 NADPH + SO_4_ → Cys + Ac | | abc + de → abc + de | |
| v48(v49) | | Asp + Pyr + Glu + SucCoA + ATP +2 NADPH → LL-DAP + AKG + Suc | | abcd + efg + hijkl + mnop → abcdgfe + hijkl + mnop | |
| v49(v50) | | LL-DAP → Lys + CO_2_ | | abcdefg → abcdef + g | |
| v50(v51) | | Asp + 2 ATP + 2 NADPH → Thr | | abcd → abcd | |
| v51(v52) | | Asp + METHF + Cys + SucCoA + ATP + 2 NADPH → Met + Pyr + Suc + NH_3_ | | abcd + e + fgh + ijkl → abcde + fgh + ijkl | |
| v52(v53) | | Pyr + Pyr + Glu + NADPH → Val + CO2 + AKG | | abc + def + ghijk → abcef + d + ghijk | |
| v53(v54) | | AcCoA + Pyr + Pyr + Glu + NADPH → Leu + CO_2_ + CO_2_ + AKG + NADH | | ab + cde + fgh + ijklm → abdghe + c + f + ijklm | |
| v54(v55) | | Thr + Pyr + Glu + ATP + NADPH → Ile + CO2 + AKG | | abcd + efg + hijkl → abfcdg + e + hijkl | |
| v55(v56) | | PEP + PEP + E4P + Glu + ATP + NADPH → Phe + CO_2_ + AKG | | abc + def + ghij + klmno → abcefghij + d + klmno | |
| v56(v57) | | PEP + PEP + E4P + Glu + ATP + NADPH → Tyr + CO_2_ + AKG + NADH | | abc + def + ghij + klmno → abcefhgij + d + klmno | |
| v57(v58) | | Ser + R5P + PEP + E4P + PEP + Gln + 3 ATP + NADPH → Trp + CO_2_ + GAP + Pyr + Glu | | abc + defgh + ijk + lmno + pqr + stuvw → abcedklmnoj + i + fgh + pqr + stuvw | |
| v58(v59) | | R5P + FTHF + Gln + Asp + 5 ATP → His + AKG + Fum + 2 NADH | | abcde + f + ghijk + lmno → edcbaf + hgijk + lmno | |
| **One-Carbon Metabolism** | | | | | |
| ST1(ST2/8) | | Reaction | | Carbon atom rearrangement | |
| v59(v60) | | MEETHF + NADH → METHF | | a → a | |
| v60(v61) | | MEETHF → FTHF + NADPH | | a → a | |
| **Oxidative Phosphorylation** | | | | | |
| ST1(ST2/8) | | Reaction | | Carbon atom rearrangement | |
| v61(v62) | | NADH + 1/2 O_2_ → 2 ATP | |  | |
| v62(v63) | | FADH2 + 1/2 O_2_ → ATP | |  | |
| **Transhydrogenation** | | | | | |
| ST1(ST2/8) | | Reaction | | Carbon atom rearrangement | |
| v63(v64) | | NADH ↔ NADPH | |  | |
| **ATP Hydrolysis** | | | | | |
| ST1(ST2/8) | | Reaction | | Carbon atom rearrangement | |
| v64(v65) | | ATP → ATP_EX | |  | |
| **Transport** | | | | | |
| ST1(ST2/8) | | Reaction | | Carbon atom rearrangement | |
| v65(v66) | | Ac → Ac_EX | |  | |
| v66(v67) | | CO_2_ → CO_2__EX | | a → a | |
| v67(v68) | | O_2__EX → O_2_ | |  | |
| v68(v69) | | NH_3__EX → NH_3_ | |  | |
| v69(v70) | | SO_4__EX → SO_4_ | |  | |
| **Biomass Formation** | | | | | |
| ST1(ST2/8) | | Reaction | | Carbon atom rearrangement | |
| v70(v71) | | | 0.488 Ala + 0.281 Arg + 0.229 Asn + 0.229 Asp + 0.087 Cys + 0.25 Glu | | |
| **CO_2_ exchange** | | | | | |
| ST1(ST2/8) | | Reaction | | Carbon atom rearrangement | |
| v71(v72) | | CO_2__unlabeled + CO_2_ → CO_2_ + CO_2__out | | a + b → a + b | |

**Table S4. Mass isotopomer distributions from *E. coli* grown in batch cultures with 12-13C-labeled glucose.**

| Strain | ST1 | ST2 | ST8 |
| --- | --- | --- | --- |
| Ala232 |  |  |  |
| m0 | 39.2 | 39.9 | 41.0 |
| m1 | 16.7 | 17.7 | 19.2 |
| m2 | 33.9 | 32.9 | 30.7 |
| m3 | 7.1 | 6.7 | 6.3 |
| m4 | 2.7 | 2.6 | 2.4 |
| Ala260 |  |  |  |
| m0 | 38.7 | 38.7 | 39.8 |
| m1 | 11.7 | 14.8 | 16.2 |
| m2 | 37.2 | 33.2 | 31.5 |
| m3 | 8.5 | 9.4 | 8.9 |
| m4 | 3.3 | 3.2 | 3.0 |
| m5 | 0.5 | 0.6 | 0.5 |
| Gly218 |  |  |  |
| m0 | 45.7 | 47.1 | 49.2 |
| m1 | 40.4 | 39.3 | 37.8 |
| m2 | 10.2 | 9.8 | 9.6 |
| m3 | 3.2 | 3.3 | 3.0 |
| Gly246 |  |  |  |
| m0 | 43.3 | 44.0 | 45.6 |
| m1 | 40.5 | 38.3 | 37.0 |
| m2 | 11.8 | 12.8 | 12.9 |
| m3 | 3.7 | 3.9 | 3.7 |
| m4 | 0.6 | 0.8 | 0.7 |
| Val260 |  |  |  |
| m0 | 20.2 | 21.2 | 22.9 |
| m1 | 13.3 | 14.5 | 16.1 |
| m2 | 33.4 | 33.0 | 31.8 |
| m3 | 13.8 | 13.7 | 13.6 |
| m4 | 14.9 | 13.7 | 12.1 |
| m5 | 3.2 | 2.8 | 2.6 |
| m6 | 1.1 | 1.0 | 0.9 |
| Val288 |  |  |  |
| m0 | 20.1 | 20.5 | 22.2 |
| m1 | 10.5 | 12.5 | 13.9 |
| m2 | 34.1 | 32.6 | 31.7 |
| m3 | 12.8 | 14.2 | 14.1 |
| m4 | 16.7 | 14.6 | 13.1 |
| m5 | 4.0 | 4.1 | 3.6 |
| m6 | 1.4 | 1.2 | 1.1 |
| m7 | 0.2 | 0.2 | 0.2 |
| Leu274 |  |  |  |
| m0 | 12.3 | 12.6 | 14.4 |
| m1 | 15.8 | 17.2 | 18.3 |
| m2 | 24.7 | 24.9 | 25.0 |
| m3 | 21.9 | 22.1 | 21.3 |
| m4 | 14.4 | 13.5 | 12.6 |
| m5 | 8.2 | 7.5 | 6.5 |
| m6 | 2.1 | 1.7 | 1.5 |
| m7 | 0.6 | 0.5 | 0.4 |
| Ile274 |  |  |  |
| m0 | 14.5 | 14.7 | 16.3 |
| m1 | 14.8 | 16.5 | 18.0 |
| m2 | 27.9 | 27.1 | 26.9 |
| m3 | 19.5 | 19.9 | 19.4 |
| m4 | 14.5 | 13.8 | 12.5 |
| m5 | 6.6 | 6.0 | 5.3 |
| m6 | 1.8 | 1.5 | 1.3 |
| m7 | 0.5 | 0.4 | 0.3 |
| Ser362 |  |  |  |
| m0 | 32.9 | 32.5 | 33.0 |
| m1 | 19.6 | 23.4 | 26.1 |
| m2 | 31.4 | 30.1 | 27.9 |
| m3 | 10.8 | 9.3 | 8.8 |
| m4 | 4.2 | 3.7 | 3.4 |
| Ser390 |  |  |  |
| m0 | 31.7 | 31.7 | 32.0 |
| m1 | 18.7 | 21.8 | 24.0 |
| m2 | 31.8 | 29.6 | 28.0 |
| m3 | 11.7 | 11.1 | 10.6 |
| m4 | 4.7 | 4.5 | 4.1 |
| m5 | 1.1 | 1.1 | 1.0 |
| Phe302 |  |  |  |
| m0 | 41.7 | 42.3 | 43.7 |
| m1 | 40.2 | 37.6 | 36.9 |
| m2 | 13.0 | 14.7 | 14.3 |
| m3 | 4.0 | 4.3 | 4.1 |
| m4 | 0.8 | 0.9 | 0.9 |
| Phe308 |  |  |  |
| m0 | 15.9 | 14.5 | 15.5 |
| m1 | 9.8 | 11.7 | 13.0 |
| m2 | 29.0 | 25.9 | 25.4 |
| m3 | 14.2 | 16.5 | 16.8 |
| m4 | 17.5 | 16.6 | 15.6 |
| m5 | 7.5 | 8.3 | 7.8 |
| m6 | 4.1 | 4.3 | 3.9 |
| m7 | 1.6 | 1.8 | 1.6 |
| m8 | 0.4 | 0.5 | 0.4 |
| Asp302 |  |  |  |
| m0 | 36.6 | 36.8 | 38.8 |
| m1 | 31.8 | 31.5 | 30.3 |
| m2 | 22.8 | 23.3 | 22.7 |
| m3 | 6.5 | 6.1 | 6.0 |
| m4 | 1.9 | 1.9 | 1.9 |
| Asp390 |  |  |  |
| m0 | 23.9 | 24.2 | 25.3 |
| m1 | 22.8 | 24.3 | 25.4 |
| m2 | 28.1 | 27.8 | 27.0 |
| m3 | 16.5 | 15.8 | 14.8 |
| m4 | 6.2 | 5.8 | 5.4 |
| m5 | 2.0 | 1.8 | 1.6 |
| Asp418 |  |  |  |
| m0 | 20.8 | 20.8 | 22.4 |
| m1 | 18.5 | 20.4 | 21.0 |
| m2 | 28.1 | 27.5 | 27.3 |
| m3 | 17.5 | 17.6 | 16.5 |
| m4 | 10.2 | 9.5 | 8.8 |
| m5 | 3.5 | 3.1 | 2.8 |
| m6 | 1.1 | 1.0 | 0.9 |
| Glu330 |  |  |  |
| m0 | 16.5 | 17.3 | 18.4 |
| m1 | 18.4 | 19.9 | 21.9 |
| m2 | 29.3 | 29.0 | 28.1 |
| m3 | 18.0 | 17.9 | 17.6 |
| m4 | 13.0 | 11.9 | 10.5 |
| m5 | 3.7 | 3.0 | 2.7 |
| m6 | 1.1 | 0.9 | 0.8 |
| Glu432 |  |  |  |
| m0 | 12.4 | 12.8 | 14.3 |
| m1 | 14.6 | 15.6 | 17.1 |
| m2 | 25.2 | 25.9 | 25.8 |
| m3 | 20.0 | 20.4 | 19.9 |
| m4 | 15.6 | 14.8 | 13.7 |
| m5 | 8.1 | 7.2 | 6.4 |
| m6 | 3.0 | 2.4 | 2.1 |
| m7 | 0.9 | 0.7 | 0.6 |
| Tyr302 |  |  |  |
| m0 | 41.7 | 42.6 | 44.2 |
| m1 | 40.8 | 38.2 | 37.2 |
| m2 | 13.3 | 14.8 | 14.5 |
| m3 | 4.1 | 4.3 | 4.1 |

**Table S5.** Results of 13C-metabolic flux analysis for *E. coli* grown in batch cultures with 12-13C-labeled glucose.

| Strain | ST1 |  |  |  |  |
| --- | --- | --- | --- | --- | --- |
| SSR | 20.1 |  |  |  |  |
| Flux No. | Eqn | Best fit | LB95 | UB95 | SD |
| 1 | Glc_EX + PEP → G6P + Pyr | 100.0 | 100.0 | 100.0 | 0.0 |
| 2 | G6P ↔ F6P (net) | 72.8 | 67.6 | 78.1 | 2.6 |
| 3 | F6P + ATP → FBP | 79.1 | 76.8 | 80.8 | 1.0 |
| 4 | FBP ↔ DHAP + GAP (net) | 79.1 | 76.8 | 80.8 | 1.0 |
| 5 | DHAP ↔ GAP (net) | 79.1 | 76.8 | 80.8 | 1.0 |
| 6 | GAP ↔ 3PG + ATP + NADH (net) | 167.0 | 164.6 | 169.4 | 1.2 |
| 7 | 3PG ↔ PEP (net) | 154.0 | 150.7 | 157.4 | 1.7 |
| 8 | PEP → Pyr + ATP | 28.1 | 23.0 | 33.8 | 2.7 |
| 9 | G6P → 6PG + NADPH | 25.6 | 20.4 | 31.0 | 2.6 |
| 10 | 6PG → Ru5P + CO_2_ + NADPH | 18.3 | 12.3 | 23.8 | 2.9 |
| 11 | Ru5P ↔ X5P (net) | 6.8 | 2.9 | 10.8 | 2.0 |
| 12 | Ru5P ↔ R5P (net) | 11.5 | 9.4 | 13.1 | 0.9 |
| 13 | X5P ↔ TKC2 + GAP (net) | 6.8 | 2.9 | 10.8 | 2.0 |
| 14 | F6P ↔ TKC2 + E4P (net) | -2.1 | -4.2 | -0.1 | 1.0 |
| 15 | S7P ↔ TKC2 + R5P (net) | -4.8 | -6.7 | -2.8 | 1.0 |
| 16 | F6P ↔ TAC3 + GAP (net) | -4.8 | -6.7 | -2.8 | 1.0 |
| 17 | S7P ↔ TAC3 + E4P (net) | 4.8 | 2.8 | 6.7 | 1.0 |
| 18 | 6PG → KDPG | 7.3 | 5.7 | 9.3 | 0.9 |
| 19 | KDPG → Pyr + GAP | 7.3 | 5.7 | 9.3 | 0.9 |
| 20 | Pyr → AcCoA + CO_2_ + NADH | 115.6 | 109.8 | 123.2 | 3.3 |
| 21 | OAC + AcCoA → Cit | 32.1 | 25.6 | 41.9 | 4.1 |
| 22 | Cit ↔ ICIT (net) | 32.1 | 25.6 | 41.9 | 4.1 |
| 23 | ICIT ↔ AKG + CO_2_ + NADPH (net) | 30.7 | 21.2 | 41.9 | 5.2 |
| 24 | AKG → SucCoA + CO_2_ + NADH | 22.6 | 12.7 | 34.5 | 5.5 |
| 25 | SucCoA ↔ Suc + ATP (net) | 19.0 | 8.9 | 31.3 | 5.6 |
| 26 | Suc ↔ Fum + FADH2 (net) | 24.0 | 17.1 | 34.5 | 4.3 |
| 27 | Fum ↔ Mal (net) | 26.8 | 20.0 | 37.0 | 4.2 |
| 28 | Mal ↔ OAC + NADH (net) | 28.3 | 23.4 | 37.4 | 3.5 |
| 29 | ICIT → Glyox + Suc | 1.5 | 0.0 | 5.6 | 1.4 |
| 30 | Glyox + AcCoA → Mal | 1.5 | 0.0 | 5.6 | 1.4 |
| 31 | Mal → Pyr + CO_2_ + NADPH | 0.0 | 0.0 | 4.7 | 1.2 |
| 32 | Mal → Pyr + CO_2_ + NADH | 0.0 | 0.0 | 4.7 | 1.2 |
| 33 | PEP + CO_2_ → OAC | 23.0 | 17.9 | 32.1 | 3.5 |
| 34 | OAC + ATP → PEP + CO_2_ | 2.9 | 0.0 | 13.1 | 3.3 |
| 35 | AcCoA ↔ Ac + ATP (net) | 56.0 | 50.0 | 62.1 | 3.0 |
| 36 | AKG + NADPH + NH_3_ → Glu | 49.1 | 43.8 | 53.1 | 2.3 |
| 37 | Glu + ATP + NH_3_ → Gln | 5.1 | 4.5 | 5.5 | 0.2 |
| 38 | Glu + ATP + 2 NADPH → Pro | 1.6 | 1.4 | 1.7 | 0.1 |
| 39 | Glu + CO_2_ + Gln + Asp + AcCoA + 5 ATP + NADPH → Arg + AKG + Fum + Ac | 2.1 | 1.9 | 2.3 | 0.1 |
| 40 | OAC + Glu → Asp + AKG | 13.7 | 12.2 | 14.8 | 0.7 |
| 41 | Asp + 2 ATP + NH_3_ → Asn | 1.7 | 1.5 | 1.9 | 0.1 |
| 42 | Pyr + Glu → Ala + AKG | 3.7 | 3.3 | 4.0 | 0.2 |
| 43 | 3PG + Glu → Ser + AKG + NADH | 8.4 | 7.5 | 9.1 | 0.4 |
| 44 | Ser ↔ Gly + MEETHF (net) | 4.7 | 4.2 | 5.1 | 0.2 |
| 45 | Gly ↔ CO_2_ + MEETHF + NADH + NH_3_ (net) | 0.4 | 0.3 | 0.6 | 0.1 |
| 46 | Thr → Gly + AcCoA + NADH | 0.0 | 0.0 | 0.4 | 0.1 |
| 47 | Ser + AcCoA + 3 ATP + 4 NADPH + SO_4_ → Cys + Ac | 1.8 | 1.6 | 1.9 | 0.1 |
| 48 | Asp + Pyr + Glu + SucCoA + ATP +2 NADPH → LL-DAP + AKG + Suc | 2.5 | 2.2 | 2.7 | 0.1 |
| 49 | LL-DAP → Lys + CO_2_ | 2.5 | 2.2 | 2.7 | 0.1 |
| 50 | Asp + 2 ATP + 2 NADPH → Thr | 3.9 | 3.5 | 4.4 | 0.2 |
| 51 | Asp + METHF + Cys + SucCoA + ATP + 2 NADPH → Met + Pyr + Suc + NH_3_ | 1.1 | 1.0 | 1.2 | 0.1 |
| 52 | Pyr + Pyr + Glu + NADPH → Val + CO2 + AKG | 3.0 | 2.7 | 3.3 | 0.1 |
| 53 | AcCoA + Pyr + Pyr + Glu + NADPH → Leu + CO_2_ + CO_2_ + AKG + NADH | 3.2 | 2.9 | 3.5 | 0.2 |
| 54 | Thr + Pyr + Glu + ATP + NADPH → Ile + CO2 + AKG | 2.1 | 1.9 | 2.2 | 0.1 |
| 55 | PEP + PEP + E4P + Glu + ATP + NADPH → Phe + CO_2_ + AKG | 1.3 | 1.2 | 1.4 | 0.1 |
| 56 | PEP + PEP + E4P + Glu + ATP + NADPH → Tyr + CO_2_ + AKG + NADH | 1.0 | 0.9 | 1.1 | 0.0 |
| 57 | Ser + R5P + PEP + E4P + PEP + Gln + 3 ATP + NADPH → Trp + CO_2_ + GAP + Pyr + Glu | 0.4 | 0.4 | 0.4 | 0.0 |
| 58 | R5P + FTHF + Gln + Asp + 5 ATP → His + AKG + Fum + 2 NADH | 0.7 | 0.6 | 0.7 | 0.0 |
| 59 | MEETHF + NADH → METHF | 1.1 | 1.0 | 1.2 | 0.1 |
| 60 | MEETHF → FTHF + NADPH | 0.7 | 0.6 | 0.7 | 0.0 |
| 61 | NADH + 1/2 O_2_ → 2 ATP | 305.4 | 271.9 | 357.7 | 21.5 |
| 62 | FADH2 + 1/2 O_2_ → ATP | 24.0 | 17.1 | 34.5 | 4.3 |
| 63 | NADH ↔ NADPH (net) | 52.2 | 20.8 | 71.3 | 12.6 |
| 64 | ATP → ATP_EX | 526.9 | 419.6 | 680.9 | 65.3 |
| 65 | Ac → Ac_EX | 59.9 | 54.0 | 65.8 | 2.9 |
| 66 | CO_2_ → CO_2__EX | 182.0 | 163.3 | 211.3 | 12.0 |
| 67 | O_2__EX → O_2_ | 164.7 | 144.7 | 195.7 | 12.7 |
| 68 | NH_3__EX → NH_3_ | 54.4 | 48.5 | 58.8 | 2.6 |
| 69 | SO_4__EX → SO_4_ | 1.8 | 1.6 | 1.9 | 0.1 |
| 70 | 0.488*Ala + 0.281*Arg + 0.229*Asn + 0.229*Asp + 0.087*Cys + 0.25*Glu | 7.5 | 6.7 | 8.1 | 0.4 |
| 71 | CO_2__unlabeled + CO_2_ → CO_2_ + CO_2__out | 0.0 | 0.0 | 45.4 | 11.4 |
| **EXCHANGE FLUXES** | | Best fit | LB95 | UB95 |  |
| 2 | G6P ↔ F6P (exch) | 0.0 | 0.0 | Inf |  |
| 4 | FBP ↔ DHAP + GAP (exch) | 7.3 | 0.0 | Inf |  |
| 5 | DHAP ↔ GAP (exch) | 0.4 | 0.0 | Inf |  |
| 6 | GAP ↔ 3PG + ATP + NADH (exch) | >1000 | 0.0 | >1000 |  |
| 7 | 3PG ↔ PEP (exch) | >1000 | 0.0 | Inf |  |
| 11 | Ru5P ↔ X5P (exch) | 51.6 | 3.1 | Inf |  |
| 12 | Ru5P ↔ R5P (exch) | 28.3 | 0.0 | Inf |  |
| 13 | X5P ↔ TKC2 + GAP (exch) | 19.0 | 3.1 | Inf |  |
| 14 | F6P ↔ TKC2 + E4P (exch) | 7.3 | 0.0 | 14.3 |  |
| 15 | S7P ↔ TKC2 + R5P (exch) | 24.5 | 0.0 | Inf |  |
| 16 | F6P ↔ TAC3 + GAP (exch) | 59.6 | 4.6 | Inf |  |
| 17 | S7P ↔ TAC3 + E4P (exch) | 3.9 | 0.0 | Inf |  |
| 22 | Cit ↔ ICIT (exch) | 0.0 | 0.0 | Inf |  |
| 23 | ICIT ↔ AKG + CO_2_ + NADPH (exch) | 2.7 | 0.0 | Inf |  |
| 25 | SucCoA ↔ Suc + ATP (exch) | 2.7 | 0.0 | Inf |  |
| 26 | Suc ↔ Fum + FADH2 (exch) | 0.4 | 0.0 | Inf |  |
| 27 | Fum ↔ Mal (exch) | 59.8 | 21.7 | Inf |  |
| 28 | Mal ↔ OAC + NADH (exch) | >1000 | 23.4 | Inf |  |
| 35 | AcCoA ↔ Ac + ATP (exch) | 4.3 | 0.0 | Inf |  |
| 44 | Ser ↔ Gly + MEETHF (exch) | 1.8 | 1.4 | 2.2 |  |
| 45 | Gly ↔ CO_2_ + MEETHF + NADH + NH_3_ (exch) | 0.0 | 0.0 | 1.0 |  |
| 63 | NADH ↔ NADPH (exch) | 14.2 | 0.0 | Inf |  |
| **FRACTIONALABELING OF AMINO ACIDS (G-VALUES)** | | Best fit | LB95 | UB95 |  |
| 1 | Fractional labeling of Ala | 98.7 | 97.0 | 100.0 |  |
| 2 | Fractional labeling of Gly | 99.0 | 95.5 | 100.0 |  |
| 3 | Fractional labeling of Val | 98.4 | 97.2 | 99.5 |  |
| 4 | Fractional labeling of Leu | 98.0 | 96.7 | 99.2 |  |
| 5 | Fractional labeling of Ile | 98.4 | 97.0 | 99.7 |  |
| 6 | Fractional labeling of Ser | 100.0 | 98.2 | 100.0 |  |
| 7 | Fractional labeling of Phe | 98.3 | 96.9 | 99.8 |  |
| 8 | Fractional labeling of Asp | 98.0 | 96.4 | 99.5 |  |
| 9 | Fractional labeling of Glu | 98.1 | 97.0 | 99.2 |  |
| 10 | Fractional labeling of Tyr | 99.1 | 96.4 | 100.0 |  |
| Strain | ST2 |  |  |  |  |
| SSR | 6.7 |  |  |  |  |
| Flux No. | Eqn | Best fit | LB95 | UB95 | SD |
| 1 | Glc_EX + ATP → Glc | 100.0 | 100.0 | 100.0 | 0.0 |
| 2 | Glc + ATP → G6P | 100.0 | 100.0 | 100.0 | 0.0 |
| 3 | G6P ↔ F6P (net) | 62.7 | 57.9 | 78.1 | 5.0 |
| 4 | F6P + ATP → FBP | 77.2 | 74.7 | 81.8 | 1.8 |
| 5 | FBP ↔ DHAP + GAP (net) | 77.2 | 74.7 | 81.8 | 1.8 |
| 6 | DHAP ↔ GAP (net) | 77.2 | 74.7 | 81.8 | 1.8 |
| 7 | GAP ↔ 3PG + ATP + NADH (net) | 161.3 | 157.5 | 167.2 | 2.4 |
| 8 | 3PG ↔ PEP (net) | 144.2 | 138.8 | 152.2 | 3.3 |
| 9 | PEP → Pyr + ATP | 115.8 | 108.5 | 124.3 | 4.0 |
| 10 | G6P → 6PG + NADPH | 35.2 | 22.4 | 40.1 | 4.4 |
| 11 | 6PG → Ru5P + CO_2_ + NADPH | 33.4 | 17.0 | 39.3 | 5.6 |
| 12 | Ru5P ↔ X5P (net) | 15.2 | 3.6 | 19.8 | 4.0 |
| 13 | Ru5P ↔ R5P (net) | 18.3 | 13.1 | 20.2 | 1.8 |
| 14 | X5P ↔ TKC2 + GAP (net) | 15.2 | 3.6 | 19.8 | 4.0 |
| 15 | F6P ↔ TKC2 + E4P (net) | (5.8) | (8.3) | (0.7) | 1.9 |
| 16 | S7P ↔ TKC2 + R5P (net) | (9.4) | (11.5) | (4.5) | 1.7 |
| 17 | F6P ↔ TAC3 + GAP (net) | (9.4) | (11.5) | (4.5) | 1.7 |
| 18 | S7P ↔ TAC3 + E4P (net) | 9.4 | 4.5 | 11.5 | 1.7 |
| 19 | 6PG → KDPG | 1.8 | 0.0 | 4.0 | 1.0 |
| 20 | KDPG → Pyr + GAP | 1.8 | 0.0 | 4.0 | 1.0 |
| 21 | Pyr → AcCoA + CO_2_ + NADH | 91.5 | 84.6 | 101.7 | 4.3 |
| 22 | OAC + AcCoA → Cit | 54.9 | 39.3 | 76.1 | 9.2 |
| 23 | Cit ↔ ICIT (net) | 54.9 | 39.3 | 76.1 | 9.2 |
| 24 | ICIT ↔ AKG + CO_2_ + NADPH (net) | 46.9 | 22.6 | 76.1 | 13.4 |
| 25 | AKG → SucCoA + CO_2_ + NADH | 36.2 | 10.7 | 67.1 | 14.1 |
| 26 | SucCoA ↔ Suc + ATP (net) | 31.6 | 5.5 | 63.1 | 14.4 |
| 27 | Suc ↔ Fum + FADH2 (net) | 44.2 | 27.3 | 67.1 | 9.9 |
| 28 | Fum ↔ Mal (net) | 47.9 | 31.4 | 70.2 | 9.7 |
| 29 | Mal ↔ OAC + NADH (net) | 55.8 | 44.5 | 70.6 | 6.5 |
| 30 | ICIT → Glyox + Suc | 8.0 | 0.0 | 18.7 | 4.7 |
| 31 | Glyox + AcCoA → Mal | 8.0 | 0.0 | 18.7 | 4.7 |
| 32 | Mal → Pyr + CO_2_ + NADPH | 0.0 | 0.0 | 6.6 | 1.6 |
| 33 | Mal → Pyr + CO_2_ + NADH | 0.0 | NaN | 6.6 | #VALUE! |
| 34 | PEP + CO_2_ → OAC | 46.2 | 36.3 | 60.4 | 6.0 |
| 35 | OAC + ATP → PEP + CO_2_ | 25.3 | 13.8 | 37.4 | 5.9 |
| 36 | AcCoA ↔ Ac + ATP (net) | (5.1) | (5.8) | (4.3) | 0.4 |
| 37 | AKG + NADPH + NH_3_ → Glu | 64.9 | 54.5 | 72.8 | 4.6 |
| 38 | Glu + ATP + NH_3_ → Gln | 6.7 | 5.6 | 7.6 | 0.5 |
| 39 | Glu + ATP + 2 NADPH → Pro | 2.1 | 1.8 | 2.4 | 0.2 |
| 40 | Glu + CO_2_ + Gln + Asp + AcCoA + 5 ATP + NADPH → Arg + AKG + Fum + Ac | 2.8 | 2.3 | 3.2 | 0.2 |
| 41 | OAC + Glu → Asp + AKG | 18.4 | 15.2 | 20.9 | 1.4 |
| 42 | Asp + 2 ATP + NH_3_ → Asn | 2.3 | 1.9 | 2.6 | 0.2 |
| 43 | Pyr + Glu → Ala + AKG | 4.8 | 4.1 | 5.5 | 0.4 |
| 44 | 3PG + Glu → Ser + AKG + NADH | 10.9 | 9.1 | 12.6 | 0.9 |
| 45 | Ser ↔ Gly + MEETHF (net) | 6.0 | 5.0 | 7.1 | 0.5 |
| 46 | Gly ↔ CO_2_ + MEETHF + NADH + NH_3_ (net) | 0.7 | 0.4 | 1.1 | 0.2 |
| 47 | Thr → Gly + AcCoA + NADH | 0.4 | 0.0 | 1.2 | 0.3 |
| 48 | Ser + AcCoA + 3 ATP + 4 NADPH + SO_4_ → Cys + Ac | 2.3 | 1.9 | 2.6 | 0.2 |
| 49 | Asp + Pyr + Glu + SucCoA + ATP +2 NADPH → LL-DAP + AKG + Suc | 3.2 | 2.7 | 3.7 | 0.2 |
| 50 | LL-DAP → Lys + CO_2_ | 3.2 | 2.7 | 3.7 | 0.2 |
| 51 | Asp + 2 ATP + 2 NADPH → Thr | 5.6 | 4.3 | 6.6 | 0.6 |
| 52 | Asp + METHF + Cys + SucCoA + ATP + 2 NADPH → Met + Pyr + Suc + NH_3_ | 1.4 | 1.2 | 1.6 | 0.1 |
| 53 | Pyr + Pyr + Glu + NADPH → Val + CO2 + AKG | 4.0 | 3.4 | 4.5 | 0.3 |
| 54 | AcCoA + Pyr + Pyr + Glu + NADPH → Leu + CO_2_ + CO_2_ + AKG + NADH | 4.2 | 3.6 | 4.8 | 0.3 |
| 55 | Thr + Pyr + Glu + ATP + NADPH → Ile + CO2 + AKG | 2.7 | 2.3 | 3.1 | 0.2 |
| 56 | PEP + PEP + E4P + Glu + ATP + NADPH → Phe + CO_2_ + AKG | 1.7 | 1.5 | 2.0 | 0.1 |
| 57 | PEP + PEP + E4P + Glu + ATP + NADPH → Tyr + CO_2_ + AKG + NADH | 1.3 | 1.1 | 1.5 | 0.1 |
| 58 | Ser + R5P + PEP + E4P + PEP + Gln + 3 ATP + NADPH → Trp + CO_2_ + GAP + Pyr + Glu | 0.5 | 0.5 | 0.6 | 0.0 |
| 59 | R5P + FTHF + Gln + Asp + 5 ATP → His + AKG + Fum + 2 NADH | 0.9 | 0.8 | 1.0 | 0.1 |
| 60 | MEETHF + NADH → METHF | 1.4 | 1.2 | 1.6 | 0.1 |
| 61 | MEETHF → FTHF + NADPH | 0.9 | 0.8 | 1.0 | 0.1 |
| 62 | NADH + 1/2 O_2_ → 2 ATP | 324.5 | 229.7 | 433.0 | 50.8 |
| 63 | FADH2 + 1/2 O_2_ → ATP | 44.2 | 27.3 | 67.1 | 9.9 |
| 64 | NADH ↔ NADPH (net) | 52.6 | (8.3) | 112.0 | 30.1 |
| 65 | ATP → ATP_EX | 303.3 | 41.7 | 637.8 | 149.0 |
| 66 | Ac → Ac_EX | (0.0) | 0.0 | 0.1 | 0.0 |
| 67 | CO_2_ → CO_2__EX | 207.1 | 154.6 | 269.2 | 28.6 |
| 68 | O_2__EX → O_2_ | 184.4 | 128.7 | 250.0 | 30.3 |
| 69 | NH_3__EX → NH3 | 71.7 | 60.4 | 81.3 | 5.2 |
| 70 | SO_4__EX → SO_4_ | 2.3 | 1.9 | 2.6 | 0.2 |
| 71 | 0.488*Ala + 0.281*Arg + 0.229*Asn + 0.229*Asp + 0.087*Cys + 0.25*Glu | 9.9 | 8.3 | 11.2 | 0.7 |
| 72 | CO_2__unlabeled + CO_2_ → CO_2_ + CO_2__out | 29.3 | 0.0 | 130.3 | 32.6 |
| **EXCHANGE FLUXES** | |  | LB95 | UB95 |  |
| 3 | G6P ↔ F6P (exch) | >1000 | 0.0 | Inf |  |
| 5 | FBP ↔ DHAP + GAP (exch) | 1.9 | 0.0 | >1000 |  |
| 6 | DHAP ↔ GAP (exch) | 6.1 | 0.0 | >1000 |  |
| 7 | GAP ↔ 3PG + ATP + NADH (exch) | >1000 | 0.0 | Inf |  |
| 8 | 3PG ↔ PEP (exch) | 12.2 | 0.0 | >1000 |  |
| 12 | Ru5P ↔ X5P (exch) | 56.6 | 2.7 | Inf |  |
| 13 | Ru5P ↔ R5P (exch) | 0.0 | 0.0 | Inf |  |
| 14 | X5P ↔ TKC2 + GAP (exch) | 47.4 | 2.7 | Inf |  |
| 15 | F6P ↔ TKC2 + E4P (exch) | 16.6 | 0.0 | 47.6 |  |
| 16 | S7P ↔ TKC2 + R5P (exch) | 42.6 | 0.0 | Inf |  |
| 17 | F6P ↔ TAC3 + GAP (exch) | 71.6 | 27.5 | >1000 |  |
| 18 | S7P ↔ TAC3 + E4P (exch) | 18.7 | NaN | Inf |  |
| 23 | Cit ↔ ICIT (exch) | 2.0 | 0.0 | Inf |  |
| 24 | ICIT ↔ AKG + CO_2_ + NADPH (exch) | 0.0 | 0.0 | Inf |  |
| 26 | SucCoA ↔ Suc + ATP (exch) | 4.0 | NaN | Inf |  |
| 27 | Suc ↔ Fum + FADH2 (exch) | 32.5 | 0.0 | Inf |  |
| 28 | Fum ↔ Mal (exch) | >1000 | 129.6 | Inf |  |
| 29 | Mal ↔ OAC + NADH (exch) | 408.0 | 127.0 | Inf |  |
| 36 | AcCoA ↔ Ac + ATP (exch) | 11.7 | 0.0 | >1000 |  |
| 45 | Ser ↔ Gly + MEETHF (exch) | 4.3 | 3.0 | 5.3 |  |
| 46 | Gly ↔ CO_2_ + MEETHF + NADH + NH_3_ (exch) | 0.2 | 0.0 | 1.9 |  |
| 64 | NADH ↔ NADPH (exch) | 20.3 | 0.0 | Inf |  |
| **FRACTIONALABELING OF AMINO ACIDS (G-VALUES)** | | Best fit | LB95 | UB95 |  |
| 1 | Fractional labeling of Ala | 100.0 | 98.2 | 100.0 |  |
| 2 | Fractional labeling of Gly | 99.2 | 95.2 | 100.0 |  |
| 3 | Fractional labeling of Val | 99.1 | 97.8 | 100.0 |  |
| 4 | Fractional labeling of Leu | 98.5 | 97.1 | 99.8 |  |
| 5 | Fractional labeling of Ile | 98.7 | 97.3 | 100.0 |  |
| 6 | Fractional labeling of Ser | 99.2 | 97.1 | 100.0 |  |
| 7 | Fractional labeling of Phe | 97.9 | 96.4 | 99.6 |  |
| 8 | Fractional labeling of Asp | 97.5 | 95.6 | 99.4 |  |
| 9 | Fractional labeling of Glu | 98.0 | 96.8 | 99.2 |  |
| 10 | Fractional labeling of Tyr | 98.6 | 95.6 | 100.0 |  |
| Strain | ST8 |  |  |  |  |
| SSR | 6.9 |  |  |  |  |
| Flux No. | Eqn | Best fit | LB95 | UB95 | SD |
| 1 | Glc_EX + ATP → Glc | 100.0 | 100.0 | 100.0 | 0.0 |
| 2 | Glc + ATP → G6P | 100.0 | 100.0 | 100.0 | 0.0 |
| 3 | G6P ↔ F6P (net) | 52.0 | 48.0 | 65.2 | 4.3 |
| 4 | F6P + ATP → FBP | 75.0 | 72.2 | 79.5 | 1.8 |
| 5 | FBP ↔ DHAP + GAP (net) | 75.0 | 72.2 | 79.5 | 1.8 |
| 6 | DHAP ↔ GAP (net) | 75.0 | 72.2 | 79.5 | 1.8 |
| 7 | GAP ↔ 3PG + ATP + NADH (net) | 160.2 | 154.8 | 169.1 | 3.6 |
| 8 | 3PG ↔ PEP (net) | 144.3 | 135.9 | 158.4 | 5.6 |
| 9 | PEP → Pyr + ATP | 123.6 | 112.7 | 135.3 | 5.6 |
| 10 | G6P → 6PG + NADPH | 46.1 | 32.6 | 50.2 | 4.4 |
| 11 | 6PG → Ru5P + CO_2_ + NADPH | 45.5 | 28.7 | 50.2 | 5.4 |
| 12 | Ru5P ↔ X5P (net) | 23.7 | 11.2 | 28.3 | 4.3 |
| 13 | Ru5P ↔ R5P (net) | 21.8 | 16.8 | 23.9 | 1.8 |
| 14 | X5P ↔ TKC2 + GAP (net) | 23.7 | 11.2 | 28.3 | 4.3 |
| 15 | F6P ↔ TKC2 + E4P (net) | (10.2) | (12.9) | (3.7) | 2.3 |
| 16 | S7P ↔ TKC2 + R5P (net) | (13.5) | (15.4) | (7.6) | 2.0 |
| 17 | F6P ↔ TAC3 + GAP (net) | (13.5) | (15.4) | (7.6) | 2.0 |
| 18 | S7P ↔ TAC3 + E4P (net) | 13.5 | 7.6 | 15.4 | 2.0 |
| 19 | 6PG → KDPG | 0.6 | 0.0 | 3.6 | 0.9 |
| 20 | KDPG → Pyr + GAP | 0.6 | 0.0 | 3.6 | 0.9 |
| 21 | Pyr → AcCoA + CO_2_ + NADH | 102.8 | 93.2 | 119.8 | 6.6 |
| 22 | OAC + AcCoA → Cit | 59.9 | 36.2 | 101.8 | 16.4 |
| 23 | Cit ↔ ICIT (net) | 59.9 | 36.2 | 101.8 | 16.4 |
| 24 | ICIT ↔ AKG + CO_2_ + NADPH (net) | 43.7 | 11.4 | 101.8 | 22.6 |
| 25 | AKG → SucCoA + CO_2_ + NADH | 33.8 | 0.0 | 95.2 | 23.8 |
| 26 | SucCoA ↔ Suc + ATP (net) | 29.4 | (5.3) | 92.3 | 24.4 |
| 27 | Suc ↔ Fum + FADH2 (net) | 50.0 | 25.1 | 95.2 | 17.5 |
| 28 | Fum ↔ Mal (net) | 53.4 | 29.1 | 97.5 | 17.1 |
| 29 | Mal ↔ OAC + NADH (net) | 66.7 | 47.1 | 96.3 | 12.3 |
| 30 | ICIT → Glyox + Suc | 16.2 | (0.0) | 30.5 | 7.6 |
| 31 | Glyox + AcCoA → Mal | 16.2 | 0.0 | 30.5 | 7.6 |
| 32 | Mal → Pyr + CO_2_ + NADPH | 0.0 | 0.0 | 11.4 | 2.9 |
| 33 | Mal → Pyr + CO_2_ + NADH | 2.9 | NaN | 11.4 | #VALUE! |
| 34 | PEP + CO_2_ → OAC | 39.9 | 29.4 | 58.0 | 7.1 |
| 35 | OAC + ATP → PEP + CO_2_ | 26.3 | 14.1 | 40.4 | 6.6 |
| 36 | AcCoA ↔ Ac + ATP (net) | (4.7) | (5.8) | (3.1) | 0.7 |
| 37 | AKG + NADPH + NH_3_ → Glu | 60.5 | 40.1 | 73.7 | 8.4 |
| 38 | Glu + ATP + NH_3_ → Gln | 6.2 | 4.1 | 7.5 | 0.8 |
| 39 | Glu + ATP + 2 NADPH → Pro | 1.9 | 1.3 | 2.3 | 0.3 |
| 40 | Glu + CO_2_ + Gln + Asp + AcCoA + 5 ATP + NADPH → Arg + AKG + Fum + Ac | 2.6 | 1.7 | 3.1 | 0.4 |
| 41 | OAC + Glu → Asp + AKG | 17.2 | 11.2 | 21.4 | 2.6 |
| 42 | Asp + 2 ATP + NH_3_ → Asn | 2.1 | 1.4 | 2.6 | 0.3 |
| 43 | Pyr + Glu → Ala + AKG | 4.5 | 3.0 | 5.4 | 0.6 |
| 44 | 3PG + Glu → Ser + AKG + NADH | 10.2 | 6.7 | 12.5 | 1.4 |
| 45 | Ser ↔ Gly + MEETHF (net) | 5.6 | 3.7 | 7.0 | 0.8 |
| 46 | Gly ↔ CO_2_ + MEETHF + NADH + NH_3_ (net) | 0.7 | 0.3 | 1.3 | 0.2 |
| 47 | Thr → Gly + AcCoA + NADH | 0.4 | 0.0 | 1.6 | 0.4 |
| 48 | Ser + AcCoA + 3 ATP + 4 NADPH + SO_4_ → Cys + Ac | 2.2 | 1.4 | 2.6 | 0.3 |
| 49 | Asp + Pyr + Glu + SucCoA + ATP +2 NADPH → LL-DAP + AKG + Suc | 3.0 | 2.0 | 3.6 | 0.4 |
| 50 | LL-DAP → Lys + CO_2_ | 3.0 | 2.0 | 3.6 | 0.4 |
| 51 | Asp + 2 ATP + 2 NADPH → Thr | 5.2 | 3.2 | 7.0 | 1.0 |
| 52 | Asp + METHF + Cys + SucCoA + ATP + 2 NADPH → Met + Pyr + Suc + NH_3_ | 1.3 | 0.9 | 1.6 | 0.2 |
| 53 | Pyr + Pyr + Glu + NADPH → Val + CO2 + AKG | 3.7 | 2.5 | 4.5 | 0.5 |
| 54 | AcCoA + Pyr + Pyr + Glu + NADPH → Leu + CO_2_ + CO_2_ + AKG + NADH | 4.0 | 2.6 | 4.8 | 0.5 |
| 55 | Thr + Pyr + Glu + ATP + NADPH → Ile + CO2 + AKG | 2.5 | 1.7 | 3.1 | 0.3 |
| 56 | PEP + PEP + E4P + Glu + ATP + NADPH → Phe + CO_2_ + AKG | 1.6 | 1.1 | 2.0 | 0.2 |
| 57 | PEP + PEP + E4P + Glu + ATP + NADPH → Tyr + CO_2_ + AKG + NADH | 1.2 | 0.8 | 1.5 | 0.2 |
| 58 | Ser + R5P + PEP + E4P + PEP + Gln + 3 ATP + NADPH → Trp + CO_2_ + GAP + Pyr + Glu | 0.5 | 0.3 | 0.6 | 0.1 |
| 59 | R5P + FTHF + Gln + Asp + 5 ATP → His + AKG + Fum + 2 NADH | 0.8 | 0.6 | 1.0 | 0.1 |
| 60 | MEETHF + NADH → METHF | 1.3 | 0.9 | 1.6 | 0.2 |
| 61 | MEETHF → FTHF + NADPH | 0.8 | 0.6 | 1.0 | 0.1 |
| 62 | NADH + 1/2 O_2_ → 2 ATP | 374.9 | 238.2 | 589.9 | 87.9 |
| 63 | FADH2 + 1/2 O_2_ → ATP | 50.0 | 25.1 | 95.2 | 17.5 |
| 64 | NADH ↔ NADPH (net) | 21.6 | (94.1) | 115.0 | 52.3 |
| 65 | ATP → ATP_EX | 442.3 | 31.2 | >1000 | 270.8 |
| 66 | Ac → Ac_EX | 0.0 | 0.0 | 0.1 | 0.0 |
| 67 | CO_2_ → CO_2__EX | 233.7 | 158.1 | 356.6 | 49.6 |
| 68 | O_2__EX → O_2_ | 212.4 | 132.4 | 342.5 | 52.5 |
| 69 | NH_3__EX → NH3 | 66.8 | 44.4 | 80.6 | 9.1 |
| 70 | SO_4__EX → SO_4_ | 2.2 | 1.4 | 2.6 | 0.3 |
| 71 | 0.488*Ala + 0.281*Arg + 0.229*Asn + 0.229*Asp + 0.087*Cys + 0.25*Glu | 9.2 | 6.1 | 11.1 | 1.3 |
|  | CO_2__unlabeled + CO_2_ → CO_2_ + CO_2__out | 172.4 | 6.9 | 883.0 | 219.0 |
| **EXCHANGE FLUXES** | | Best fit | LB95 | UB95 |  |
| 2 | G6P ↔ F6P (exch) | >1000 | NaN | >1000 |  |
| 4 | FBP ↔ DHAP + GAP (exch) | 4.4 | 0.0 | >1000 |  |
| 5 | DHAP ↔ GAP (exch) | 0.0 | 0.0 | >1000 |  |
| 6 | GAP ↔ 3PG + ATP + NADH (exch) | >1000 | 0.0 | Inf |  |
| 7 | 3PG ↔ PEP (exch) | 105.5 | 0.0 | Inf |  |
| 11 | Ru5P ↔ X5P (exch) | 93.6 | 0.2 | Inf |  |
| 12 | Ru5P ↔ R5P (exch) | 1.9 | 0.0 | Inf |  |
| 13 | X5P ↔ TKC2 + GAP (exch) | 29.0 | 0.1 | Inf |  |
| 14 | F6P ↔ TKC2 + E4P (exch) | 9.4 | 0.0 | 42.5 |  |
| 15 | S7P ↔ TKC2 + R5P (exch) | >1000 | 0.0 | Inf |  |
| 16 | F6P ↔ TAC3 + GAP (exch) | 69.9 | 4.5 | 446.0 |  |
| 17 | S7P ↔ TAC3 + E4P (exch) | 16.1 | NaN | Inf |  |
| 22 | Cit ↔ ICIT (exch) | 0.1 | 0.0 | Inf |  |
| 23 | ICIT ↔ AKG + CO_2_ + NADPH (exch) | 0.0 | 0.0 | Inf |  |
| 25 | SucCoA ↔ Suc + ATP (exch) | 5.3 | 0.0 | >1000 |  |
| 26 | Suc ↔ Fum + FADH2 (exch) | 35.3 | 0.0 | NaN |  |
| 27 | Fum ↔ Mal (exch) | >1000 | NaN | Inf |  |
| 28 | Mal ↔ OAC + NADH (exch) | 558.7 | 16.1 | Inf |  |
| 35 | AcCoA ↔ Ac + ATP (exch) | 1.1 | 0.0 | >1000 |  |
| 44 | Ser ↔ Gly + MEETHF (exch) | 5.6 | 3.2 | 7.7 |  |
| 45 | Gly ↔ CO_2_ + MEETHF + NADH + NH_3_ (exch) | 0.0 | 0.0 | 3.2 |  |
| 63 | NADH ↔ NADPH (exch) | 34.6 | 0.0 | >1000 |  |
| **FRACTIONALABELING OF AMINO ACIDS (G-VALUES)** | | Best fit | LB95 | UB95 |  |
| 1 | Fractional labeling of Ala | 99.0 | 96.7 | 100.0 |  |
| 2 | Fractional labeling of Gly | 98.0 | 93.0 | 100.0 |  |
| 3 | Fractional labeling of Val | 97.5 | 96.1 | 98.9 |  |
| 4 | Fractional labeling of Leu | 96.8 | 95.4 | 98.2 |  |
| 5 | Fractional labeling of Ile | 96.9 | 95.3 | 98.5 |  |
| 6 | Fractional labeling of Ser | 96.7 | 94.3 | 99.2 |  |
| 7 | Fractional labeling of Phe | 96.9 | 95.2 | 98.7 |  |
| 8 | Fractional labeling of Asp | 95.6 | 93.2 | 98.2 |  |
| 9 | Fractional labeling of Glu | 96.1 | 94.8 | 97.5 |  |
| 10 | Fractional labeling of Tyr | 96.9 | 93.6 | 100.0 |  |

Shown are the estimated net and exchange fluxes (normalized to glucose uptake rate of 100).

Accurate 95% confidence intervals and standard deviations(SD) of fluxes (LB95 = lower bound, UB95 = upper bound) were determined by evaluating the sensitivity of the minimized SSR to flux variations (Antoniewicz et al., 2006)

**
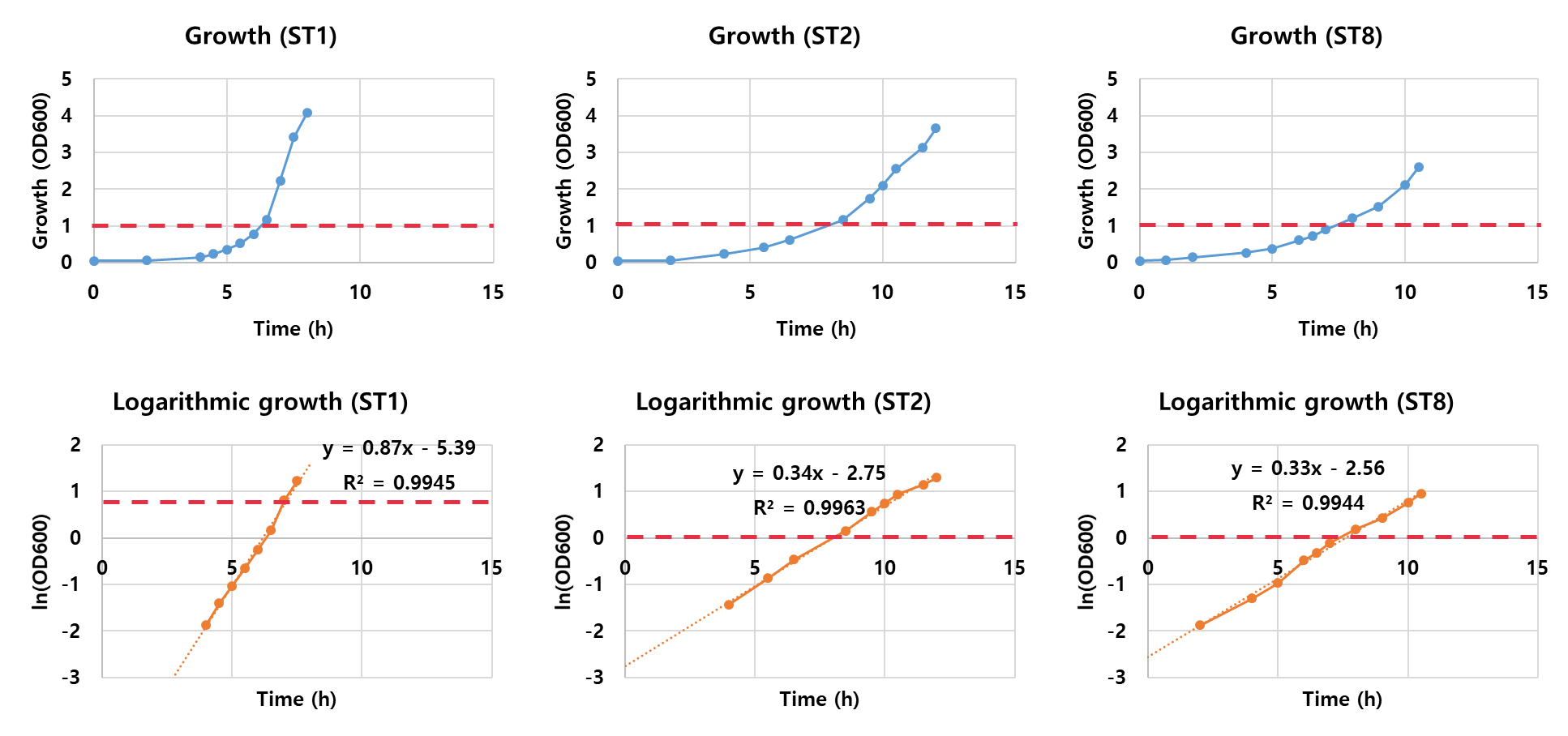
**

**Figure S1.** The growth curve of ST1, ST2, and ST8. For searching the exponentially growing section of three strains, we took a natural logarithm to the measured absorbance. The red dashed line indicates the point that OD_600_ is 1 (i.e. ln[OD_600_]=0). The bacterial growth was engaged in exponential phase around the point where OD_600_ is 1 (i.e. ln[OD_600_]=0) which is proven by well fitted linear trend line. The samples for transcriptome analysis and ^13^C MFA were acquired when OD_600_ is around 1. The specific growth rate of ST1, ST2 and ST8 were shown as the slope of trend line in logarithmic graphs.

**
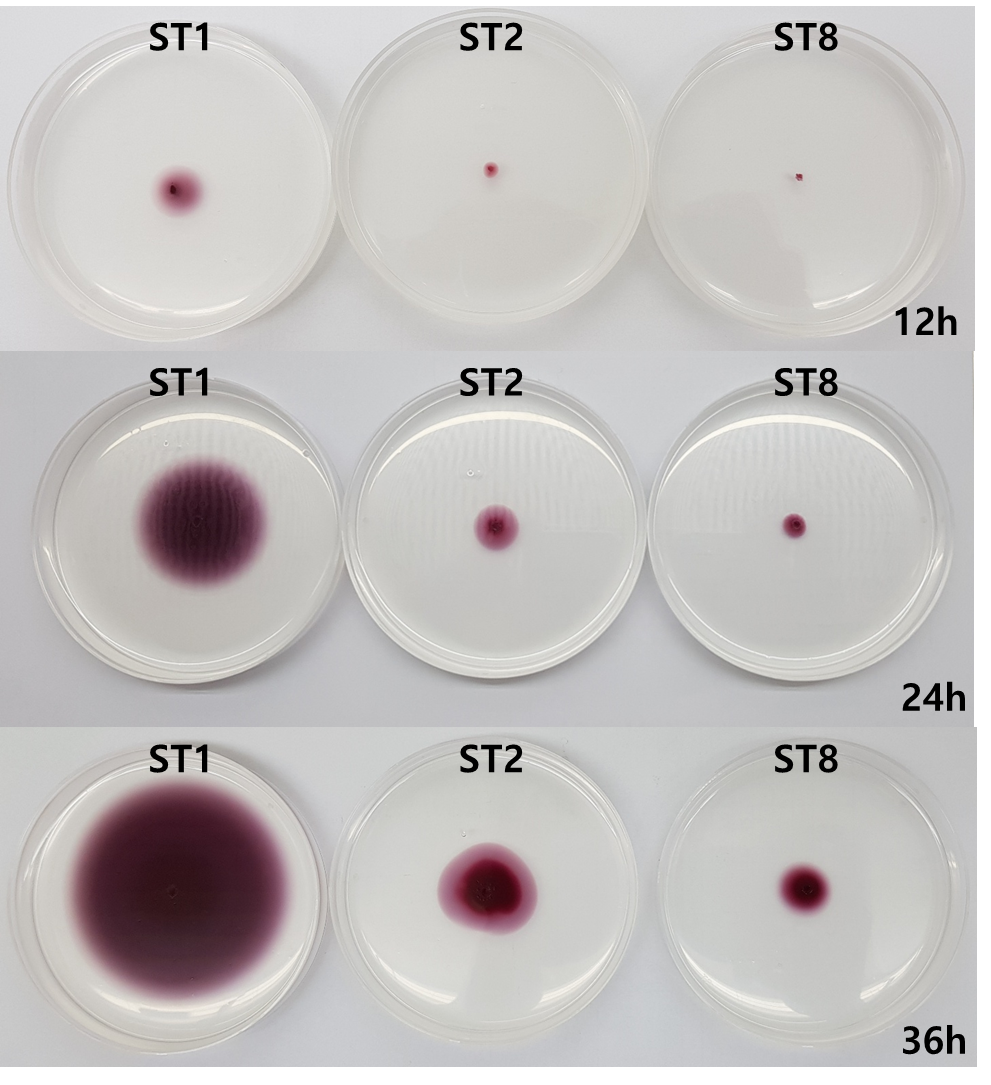
**

**Figure S2.** Motility of ST1, ST2 and ST8 was tested in M9 semi-solid agar. The fermentation broth of wild type (ST1) and mutant (ST2 and ST8) was injected by stabbing to the middle of plate and incubated in 37℃. For visualizing growth of strains, 0.05% of Triphenyltetrazolium chloride (TTC) was supplied to medium. As the cultivation progresses, ST1 rapidly spread out on the medium but it was not seen in ST2 and ST8.

**
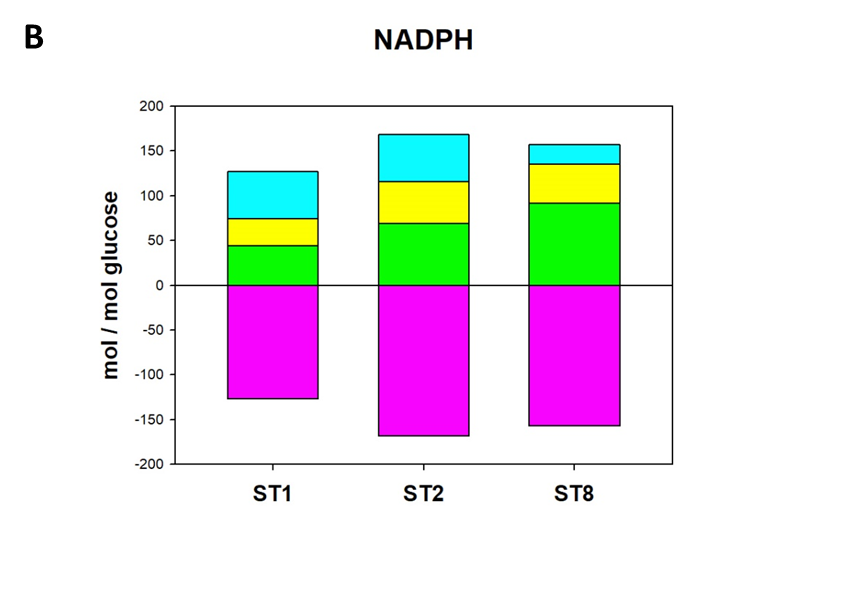
**
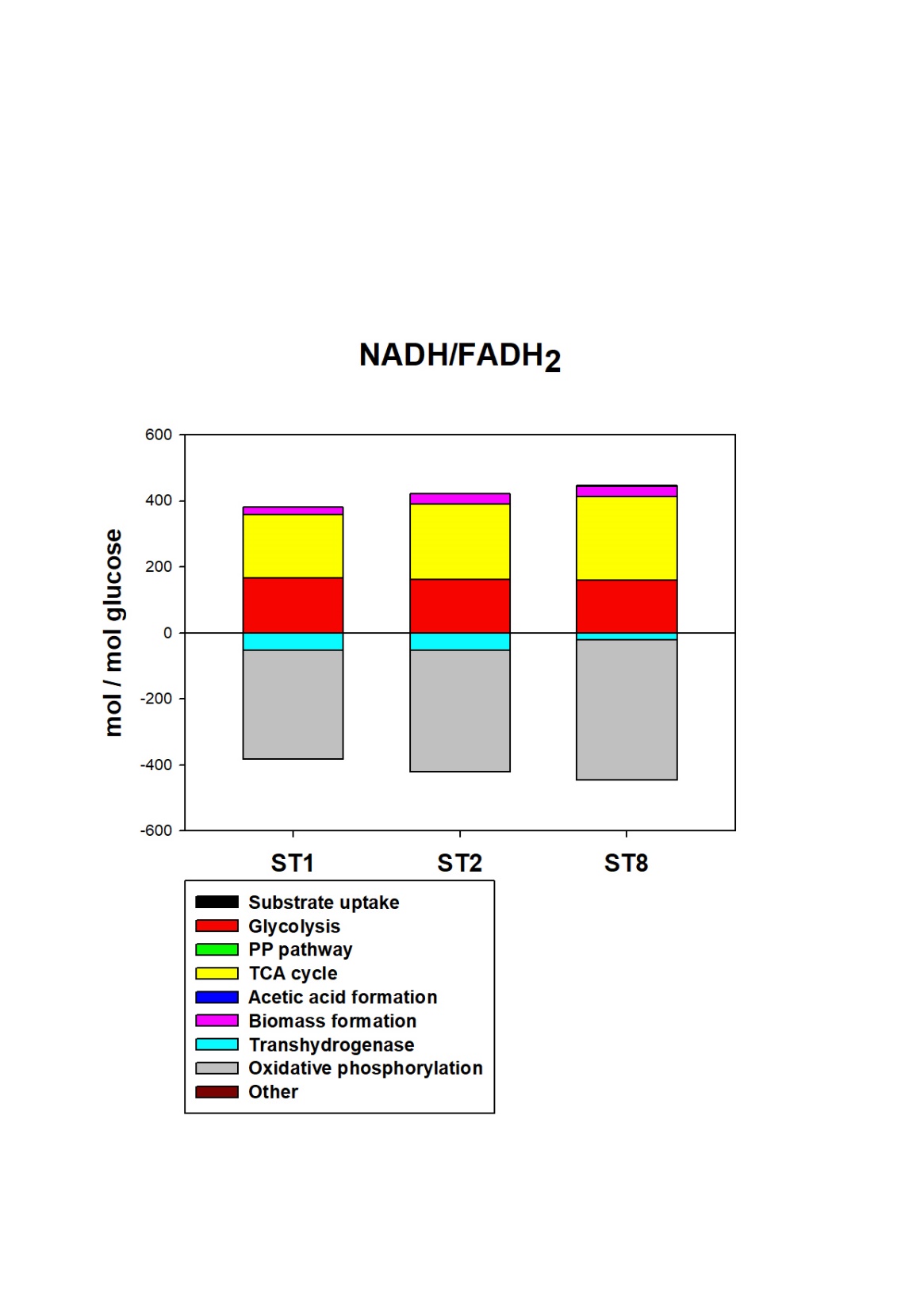
**
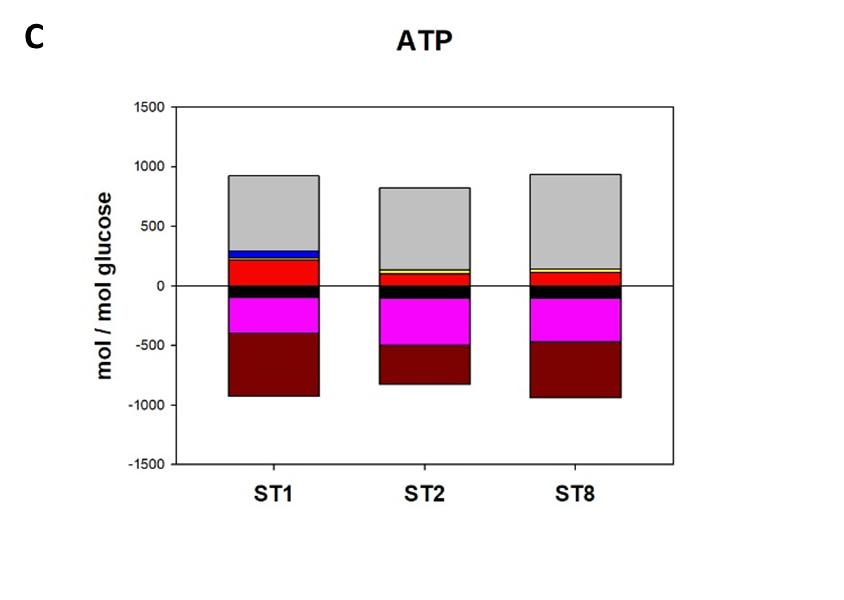
**
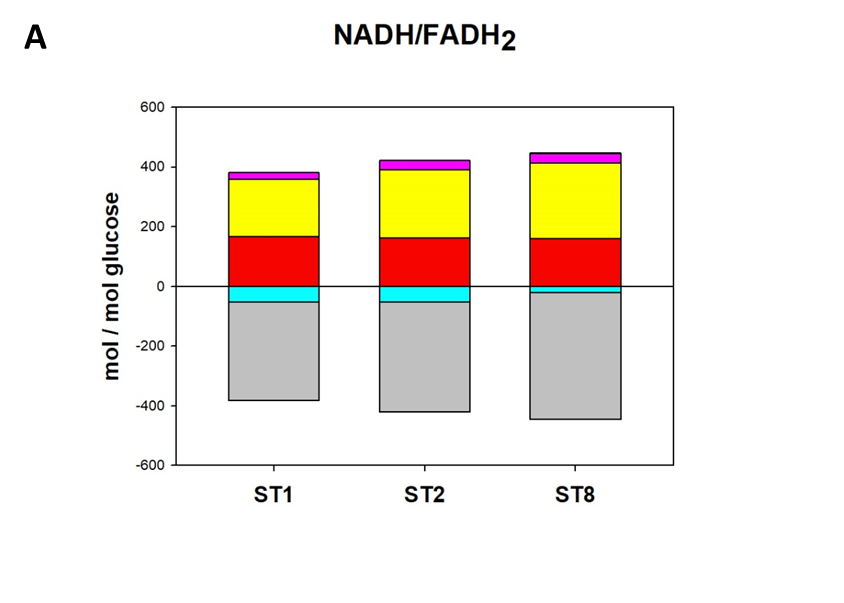


**Figure S3.** Normalized cofactor balance based on ^13^C MFA results. Shown are balances for (A) NADH/FADH_2_, (B) NADPH, and (C) ATP. The contributions of central metabolic pathways to the generation (above 0) and consumption (below 0) of cofactors were presented. We assumed P/O ratio = 2.0 (Gonzalez et al., 2017)

**References**

Antoniewicz, Maciek R., Joanne K. Kelleher, and Gregory Stephanopoulos. "Determination of confidence intervals of metabolic fluxes estimated from stable isotope measurements." Metabolic engineering 8.4 (2006): 324-337.

Gonzalez, Jacqueline E., Christopher P. Long, and Maciek R. Antoniewicz. "Comprehensive analysis of glucose and xylose metabolism in Escherichia coli under aerobic and anaerobic conditions by 13C metabolic flux analysis." Metabolic engineering 39 (2017): 9-18.
